# TNM-Accountable Whole-Body 3-Dimensional Fluorodeoxyglucose Positron Emission Tomography/Computed Tomography Report Drafting in Lung Cancer Cohorts via Structured Impressions and Organ-wise Exemplar Synthesis

**DOI:** 10.34133/research.1343

**Published:** 2026-07-21

**Authors:** Hang Wang, Xingyu Xie, Entao Liu, Yunlong Gao, Xiaorui Wu, Shan Cong, Jidong Han, Na Zhang, Hairong Zheng, Dong Liang, Xiaohui Yao, Lei Jiang, Zhanli Hu

**Affiliations:** ^1^ The Research Center for Medical AI, Shenzhen Institutes of Advanced Technology, Chinese Academy of Sciences, Shenzhen 518055, China.; ^2^ University of Chinese Academy of Sciences, Beijing 100049, China.; ^3^ The Key Laboratory of Biomedical Imaging Science and System, Chinese Academy of Sciences, State Key Laboratory of Biomedical Imaging Science and System, Shenzhen, China.; ^4^ College of Intelligent Systems Science and Engineering, Harbin Engineering University, Harbin 150001, Heilongjiang, China.; ^5^ PET Center, Department of Nuclear Medicine, Guangdong Provincial People’s Hospital (Guangdong Academy of Medical Sciences), Southern Medical University, Guangzhou, China.

## Abstract

Whole-body ^18^F-fluorodeoxyglucose positron emission tomography/computed tomography (^18^F-FDG PET/CT) is central to lung cancer staging, yet report drafting remains time intensive and error sensitive, as a single mislocalized finding can alter TNM stage and treatment decisions. We cast PET/CT report drafting as TNM-accountable decision support and propose RIDE, a 2-stage dual-modality 3-dimensional framework that separates TNM-critical anchoring from narrative completion. Stage I generates TNM-oriented structured impressions from paired PET and CT volumes; stage II performs hierarchical organ-wise exemplar retrieval and prompts a large language model to synthesize complete Findings and Impression conditioned on these inspectable artifacts. We curate a multicenter whole-body FDG PET/CT cohort of 1,583 patients from 3 hospitals, all referred for suspected or confirmed lung cancer, and evaluate it on a held-out test set of 520 cases, including 276 external cases from 2 independent institutions. RIDE achieves the strongest overall drafting performance and cross-site generalization, improving a clinically grounded competency metric by +20.4 on the external set and maintaining high clinician ratings under shift (mean Likert 4.00 internal; 4.17 external) while also delivering the best TNM staging performance among evaluated baselines. These findings support workflow-decomposed, TNM-accountable PET/CT drafting as a promising decision-support approach for human verification, and prospective studies will be valuable for further characterizing its utility in real-world clinical workflows.

## Introduction

Whole-body ^18^F-fluorodeoxyglucose positron emission tomography/computed tomography (^18^F-FDG PET/CT) is a cornerstone of oncologic imaging, providing integrated metabolic and anatomical evidence for diagnosis, staging, and treatment–response assessment [1]. In routine nuclear medicine practice, a substantial proportion of referrals involve suspected or known lung cancer, where TNM staging directly determines therapeutic strategy and prognosis: 5-year survival ranges from >70% for stage I to <10% for stage IV lung cancer [2]. PET/CT is uniquely valuable in this setting because it enables whole-body surveillance for metastatic disease while preserving anatomical location on CT. However, clinical value comes with operational burden: interpretation requires exhaustive navigation of volumetric whole-body images, reconciliation of uptake patterns with anatomy, and structured documentation that often includes quantitative descriptors such as lesion size and maximum standardized uptake value (SUV_max_) [3]. These characteristics make PET/CT reporting an attractive target for artificial-intelligence-assisted drafting but only when generated drafts remain clinically accountable and easy to verify rather than merely fluent [4].

Recent works on radiology report generation can be broadly grouped into 4 methodological families, which have substantially improved the fluency, controllability, and provenance of report drafts. First, end-to-end vision–language models (VLMs), which are mostly developed and benchmarked on 2-dimensional (2D) radiographs, have pushed the field toward scalable training and fluent report drafting, but they remain vulnerable to hallucinated or mislocalized findings [5,6]. This limitation is particularly consequential for PET/CT, where a single misspecified lesion or site may change TNM stage and downstream management [7,8]. Second, structure- or knowledge-guided approaches introduce intermediate representations (e.g., predicted labels, observation/knowledge graphs, or explicit planning) to improve consistency and controllability [9]. However, in many existing approaches, these intermediates are generic (e.g., “abnormal lung opacity”) rather than staging aligned (e.g., “T2 primary tumor in RUL with SUV_max_ 8.5”), thereby providing coarse controls and only partially covering the TNM decision schema for whole-body PET/CT, leaving gaps in TNM-critical evidence coverage and undermining staging accountability [10–13]. Third, retrieval-augmented generation and exemplar-based drafting improve controllability and provenance by conditioning generation on retrieved evidence [14,15]. Yet reliability can be limited when retrieval is performed at the whole-case or whole-report level: retrieved exemplars may match reporting style more than lesion- and organ-specific metabolic–anatomic correspondence, and domain-mismatched retrieval can inject plausible but incorrect staging cues [16,17]. Fourth, natural language processing (NLP)/large language model (LLM) studies increasingly infer or extract TNM stage from existing free-text CT/PET/CT reports, demonstrating the feasibility of text-based staging [18,19]. Still, this line of work is inherently post hoc: it does not address the unmet need in precision oncology for a staging-aware PET/CT drafting framework whose TNM conclusions are grounded in image-derived evidence rather than inferred from an already-written report [20–22]. Meanwhile, emerging volumetric CT report generation and early 3-dimensional (3D) PET/CT report-generation efforts highlight growing interest in 3D multimodal reporting [7,23,24], yet they largely emphasize descriptive findings and do not treat TNM staging as a first-class, image-grounded output for whole-body PET/CT decision support [25,26]. Together, these observations motivate a PET/CT-specific drafting workflow that (a) explicitly anchors TNM-critical content to inspectable evidence and (b) supports whole-body, organ-wise coverage while maintaining a clinically coherent narrative.

To bridge this gap, we cast whole-body 3D PET/CT report generation as structured decision support with accountable drafting. Specifically, we define TNM accountability as a practical property of the draft: staging-critical statements about (a) the primary tumor, (b) thoracic nodal disease, and (c) distant metastasis are traceable to explicit, inspectable system outputs (e.g., spatial coordinates, standardized anatomical terminologies, and metabolic uptake grades) to enable efficient human verification. To satisfy this requirement, we propose RIDE, a 2-stage framework that first generates TNM-oriented structured impressions and then performs deliberative organ-wise exemplar synthesis to draft whole-body 3D FDG PET/CT reports. In stage I, a dual-channel 3D convolutional neural network–transformer encoder learns fused metabolic–anatomical representations from PET and CT and produces a rapid, TNM-oriented structured-impression scaffold (including staging cues and quantification ranges) with broad coverage of staging-relevant attributes. In stage II, the scaffold acts as an explicit plan for whole-body reporting: it drives hierarchical, organ-wise retrieval of exemplar slots from a curated library and constrains an LLM to synthesize complete findings and impression conditioned only on the scaffold and retrieved evidence. Importantly, stage II is prompted not to mechanically copy stage I outputs; instead, conditioned on the stage I structured impression and retrieved organ-wise exemplars, it synthesizes the final narrative by prioritizing image-derived cues and using evidence-consistent, uncertainty-aware phrasing when findings are ambiguous. This design is intended to mitigate overconfident propagation of stage I errors while preserving report readability. By constraining drafting to explicit, inspectable intermediate artifacts (TNM-oriented structured impressions and organ-wise exemplars) and making the sources of staging-critical statements reviewable, the framework is designed to narrow the PET/CT “utility gap” by supporting TNM-accountable verification rather than unconstrained free-form narration.

Experimental results on a multicenter whole-body FDG PET/CT cohort with substantial out-of-institution validation (*n* = 520, external *n* = 276) demonstrate that our dual-modality (PET+CT) 3D image-to-report framework (RIDE) produces clinically strong report drafts with stable cross-site generalization. Quantitatively, our method consistently outperforms representative mainstream vision–language baselines on surface-form natural language generation (NLG) metrics, with the gain peaking at BLEU-4 (0.173 vs. 0.148 for the best prior baseline [Qwen3-VL]; ~17% relative), and shows larger margins on clinically grounded utility metrics (clinical report competency matrix [CRCM]: average margin +20.4 percentage points on the external test set). In a clinician reader study, where physicians scored all RIDE cases and additionally blind-evaluated 2 representative baselines on a subset (*n* = 223), our drafts remain highly rated under site shift (mean Likert 4.00 internal; 4.17 external), and heterogeneous LLM judges also rank our method first with a stable lead (mean +0.76 internal; +0.70 external). The 2-stage design further enables explicit, image-grounded TNM staging within the generated drafts and achieves the highest staging accuracy among the evaluated baselines (e.g., MedGemma [27], MedDr [28], RadFM [29], Qwen-based VLMs [30], and LLaVA-Med) [31]. Overall, these results indicate that RIDE narrows the PET/CT “utility gap” by improving the clinical usability of report drafts and strengthening the reliability of TNM-relevant conclusions under realistic cross-site shift. In summary, this study advances staging-aware report drafting for precision oncology in 3 ways. (a) We introduce a clinically motivated formulation in which TNM serves as a decision-level anchor for whole-body FDG PET/CT drafting. (b) We develop RIDE, a 2-stage, dual-modality 3D framework that couples TNM-oriented structured impressions with hierarchical organ-wise exemplar retrieval and deliberative synthesis, producing whole-body drafts grounded in explicit, inspectable intermediate outputs. (c) We demonstrate clinical viability through multicenter validation and physician evaluation focused on TNM correctness. To the best of our knowledge, this work is the first to draft whole-body 3D FDG PET/CT reports with an explicit TNM staging assessment as a first-class, image-grounded output. By providing a reviewable interface between multimodal imaging evidence and standardized clinical narratives, our approach is intended to support human-in-the-loop verification in PET/CT reporting workflows. Nevertheless, the present study is retrospective, and the real-world clinical utility of the framework remains to be evaluated in prospective settings. In addition, the current framework is specialized to lung cancer staging, and its broader applicability warrants further study.

## Results and Discussion

### Dataset overview and distribution statistics

We assembled a paired multicenter whole-body ^18^F-FDG PET/CT report cohort (cases collected between 2022 and 2025) from 3 institutions: Guangdong Provincial People’s Hospital (center A; *n* = 1,307), Maoming People’s Hospital (center B; *n* = 152), and the Fifth Affiliated Hospital of Sun Yat-sen University (center C; *n* = 124). Each case contains a whole-body PET volume, its corresponding CT volume, and the associated clinical report, enabling supervised learning and evaluation for image-to-report drafting as well as report-derived staging attributes. All 1,583 patients in the present cohort were referred for suspected or confirmed lung cancer. Accordingly, the current training and evaluation of RIDE are limited to lung cancer PET/CT reporting and TNM staging.

**Fig. 1.**
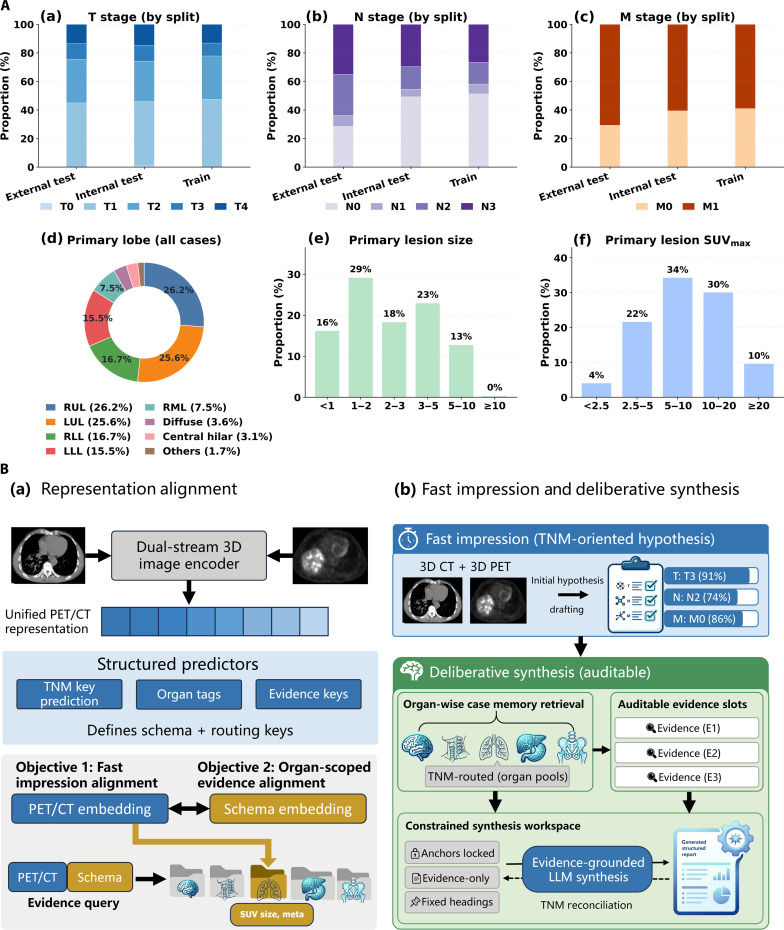
Cohort composition and framework overview. (A) Cohort composition and label/attribute distributions. The cohort represents realistic lung cancer staging with a balanced T-stage distribution, approximately 60% nodal involvement (N+), and 40% metastatic disease (M1). (a to c) Proportions of T, N, and M stages in the training, internal test, and external test splits. (d) Distribution of primary tumor lobe locations. (e) Distribution of primary-lesion size categories. (f) Distribution of primary-lesion maximum standardized uptake value (SUV_max_) categories. RUL, right upper lobe; LUL, left upper lobe; RLL, right lower lobe; LLL, left lower lobe; RML, right middle lobe. (B) Overview of the 2-stage TNM-accountable positron emission tomography/computed tomography (PET/CT) drafting framework. (a) A dual-stream 3-dimensional (3D) encoder learns unified PET/CT representations and supports routed retrieval representations via structured predictors and alignment objectives. (b) A fast pass produces a TNM-oriented structured impression, followed by deliberative organ-wise synthesis with auditable evidence slots to generate the final PET/CT report.

We adopt a site-held-out evaluation protocol to reflect realistic cross-institution deployment. To rigorously assess generalization, we reserved centers B and C (*n* = 276) exclusively for external testing with no patient or institutional overlap with training data. We summarize cohort composition and label/attribute distributions in Fig. 1A to contextualize task difficulty and reporting requirements. The distributions of T/N/M stages are reported for each split (Fig. 1A (a) to (c)). Given the dominant lung cancer setting, we further characterize the primary location of the tumor at the lobar level (Fig. 1A (d)): the right and left upper lobes account for 26.2% and 25.6% of cases, respectively (~51.8% combined). Quantitative descriptors are prevalent and can be reliably recovered from reports: primary-lesion size is extractable in 97.6% of cases and SUV_max_ in 80.6% via rule-based parsing (Fig. 1A (e) and (f)). The extracted bins show that primary-lesion size is most frequently within 1 to 2 and 3 to 5 (29% and 23%), while SUV_max_ concentrates in 5 to 10 and 10 to 20 (34% and 30%). Collectively, these cohort statistics motivate our design choice to explicitly encode TNM-relevant structure and quantification cues in stage I impressions and to enforce organ-wise coverage in downstream drafting.

### Evaluation metrics and grading protocol

To evaluate our report-drafting framework comprehensively, we adopt a dual-metric approach that jointly assesses linguistic fidelity and clinical accuracy.

#### Linguistic and clinical competency metrics

We evaluate generated reports using 2 categories of metrics. First, we use standard NLG metrics such as BLEU, METEOR, ROUGE-L, and CIDEr, to quantify surface-level similarity between generated drafts and reference reports. Second, because these metrics do not directly assess TNM-relevant clinical correctness, we introduce the CRCM, which comprises 3 unit-level scores: (a) metabolic detection score (MDS), measuring whether abnormal FDG uptake is correctly identified; (b) lesion localization score (LLS), measuring whether findings are mapped to the correct anatomical site; and (c) malignancy classification score (MCS), measuring whether the reported risk category is clinically consistent with the reference annotation. Together, these complementary metrics provide a unified evaluation. The detailed mathematical formulations for the proposed CRCM are elaborated in the “Formulation of the CRCM” section, and the standard NLG equations are provided in Section S11.

#### Grading protocol

Two senior nuclear medicine physicians independently graded the generated reports using a 5-point Likert rubric on 2 clinically oriented dimensions, accuracy and completeness, as part of a multicenter reader study spanning 3 hospitals (1 internal center and 2 external centers). Given the substantial burden associated with expert review of 3D multimodal whole-body scans, we adopted a tiered evaluation strategy. Physicians evaluated all 520 test cases for the proposed RIDE system, while a senior radiologist with more than 10 years of PET/CT interpretation experience additionally performed blinded scoring on a randomly sampled subset of 223 cases for 2 representative strong baselines (Qwen3-VL and Med3DVLM) using the same rubric. For the remaining baselines, evaluation relied on automated metrics and LLM-based judges; these comparisons are therefore interpreted as supportive automated assessments rather than direct human-validated clinical comparisons.

Accuracy is TNM driven (60%; reference or inferable TNM as ground truth, allowing ≤1 T substage difference and ≤1 N level difference, while M0/M1 must match) and additionally assesses factual correctness across 21 anatomical regions (40%). For TNM-driven grading, the reference TNM stage is taken from the original clinical report. Completeness emphasizes coverage of the 21 regions mentioned in the reference report. Scores are anchored as follows: 5, near-perfect with no clinically meaningful errors or omissions; 4, minor issues within predefined tolerance; 3, moderate issues but major staging remains recoverable (no M misclassification); 2, major staging-relevant errors or critical omissions; and 1, clinically unusable. The full 21-region checklist and the structured-element checklist used in physician grading are provided in Table S2.

As a secondary, scalable check, we use multiple LLM judges (Claude-haiku-4-5 [32], Gemini-3-pro-preview [33], GPT-o4-mini-2025-04-16 [34], and Deepseek-reasoner [35]) to comparatively score all methods under the same rubric, using a standardized prompting and automated processing pipeline to ensure consistent grading criteria. We aggregate LLM scores to reduce dependence on any single judge. Because Likert ratings are ordinal, we quantify associations using Spearman’s ρ with bootstrap confidence intervals (CIs) and report mean absolute error and mean bias (LLM minus human) to characterize alignment and grading strictness.

### Baselines

We compared against a diverse set of strong VLM baselines, including MedGemma [27], RadFM-3D [29], Qwen2.5-VL-7B [30], LLaVA-Med [31], and Med3DVLM [25]. For 2D VLM baselines (Qwen-based VLMs, LLaVA-Med, and MedDr), inputs were standardized to a fixed 2D budget: 10 key coronal slices extracted from a PET/CT concatenated representation, chosen to maximize the coverage of staging-relevant thoracic and common metastatic patterns under slice-based constraints. This protocol makes the information exposure explicit and controlled for 2D models, enabling fair comparison under realistic modality limitations. All methods were evaluated on the same report targets and under the same evaluation pipeline.

### PET/CT drafting performance with external robustness

**Fig. 2.**
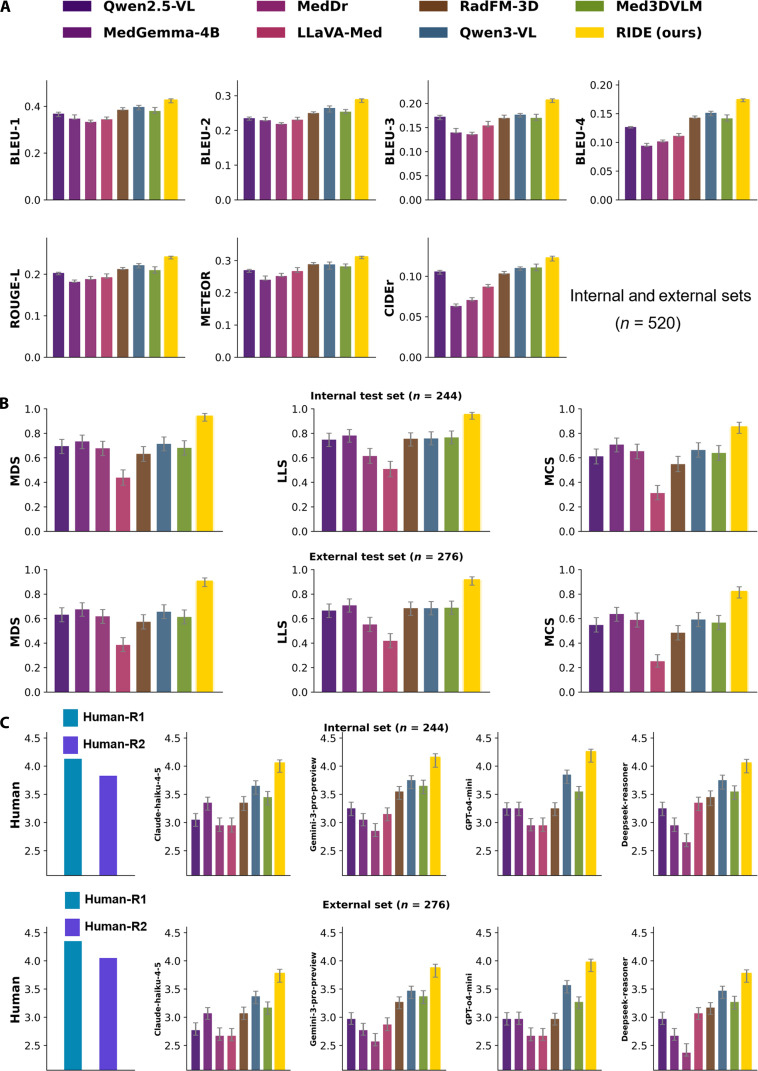
Comprehensive evaluation of positron emission tomography/computed tomography (PET/CT) report-generation performance. (A) Report-generation quality on the combined held-out test set (internal + external, *n* = 520), evaluated by conventional surface-form natural language generation (NLG) metrics (BLEU-1 to BLEU-4, ROUGE-L, METEOR, and CIDEr). (B) Clinical accountability evaluated by clinical report competency matrix (CRCM) competencies (metabolic detection score [MDS], lesion localization score [LLS], and malignancy classification score [MCS]) on the internal (*n* = 244) and external (*n* = 276) test sets. (C) Preference scores (mean 1-to-5 scale) assigned by multiple large language model (LLM) judges (Claude-haiku-4-5, Gemini-3-pro-preview, GPT-o4-mini, and Deepseek-reasoner) across the internal and external sets. Notably, 2 expert nuclear medicine physicians manually rated all 520 reports generated exclusively by our RIDE framework; their mean scores are displayed as the fixed “human” reference bars to benchmark the LLM evaluations. Error bars denote case-level bootstrap 95% confidence intervals computed from 1,000 resamples.

Across the held-out multicenter test set (*n* = 520), which includes an out-of-institution component from 2 independent institutions (*n* = 276), RIDE achieves the strongest overall report-drafting performance (Fig. 2). Improvements are consistent across conventional surface-form NLG metrics (BLEU-1/2/3/4, ROUGE-L, METEOR, and CIDEr), indicating closer alignment to reference reports across multiple granularities. Notably, the gain strengthens with BLEU order and peaks at BLEU-4 (0.173 vs. 0.148 for the best prior baseline [Qwen3-VL]; improving by 17%), suggesting more faithful composition of clinically meaningful multiword phrases rather than merely matching frequent unigrams. We also observe higher ROUGE-L, METEOR, and CIDEr, supporting improved coverage and specificity of salient clinical descriptions.

However, overlap-based NLG metrics alone cannot certify staging-critical clinical correctness in whole-body FDG PET/CT. To bridge this gap, we further summarize clinical utility using the CRCM. Across all 3 axes, RIDE achieves the best performance on both internal and out-of-institution cohorts (93.7/95.1/85.0 internal; 90.2/91.4/81.8 external for MDS/LLS/MCS), and the lead widens under external shift: relative to the strongest baseline, the average margin increases from +17.1 points internally to +20.4 points externally (Fig. 2B). The most pronounced gains appear in LLS (external +20.6 points), the aspect most vulnerable to cross-site variation in anatomical granularity and reporting style, indicating fewer plausible-but-mislocalized statements that can invalidate staging-critical content. In parallel, the consistently higher MCS (external +18.0 points) suggests more stable malignancy risk stratification aligned with clinical impression logic.

To provide an at-a-glance summary beyond automatic metrics, we report judge-based evaluations (Fig. 2C). In a clinician reader study, 2 nuclear medicine physicians rate our drafts highly on the same 5-point rubric, with strong scores on both the internal cohort (accuracy/completeness: 4.13/3.86; mean 4.00) and the out-of-institution cohort (4.25/4.09; mean 4.17), indicating that clinical utility does not degrade under site shift. As a scalable complement applied uniformly across all compared systems, 4 heterogeneous LLM judges (Claude-haiku-4-5, Gemini-3-pro-preview, GPT-o4-mini, and Deepseek-reasoner) consistently rank our method first on both internal and external cohorts, with a stable lead over the strongest competing baseline (mean +0.76 internal and +0.70 external on the same Likert scale). Together, the automated metrics and the sampled blinded physician evaluation support the conclusion that the proposed framework improves report utility in a manner aligned with TNM-focused clinical verification. In particular, on the sampled subset (*n* = 223), RIDE remained preferred by physicians over 2 representative strong baselines under blind evaluation. For the remaining baselines not included in physician scoring, cross-method comparisons rely on automated assessment and are interpreted more cautiously. Finally, we emphasize that our evaluation intentionally couples surface-form similarity with PET/CT-specific clinical utility and expert judgment: whole-body PET/CT drafting is particularly sensitive to errors in lesion localization and staging-relevant assertions, where a single plausible but incorrect statement can alter TNM stage and downstream management. In this setting, conventional NLG metrics are informative for fluency and content overlap, while the CRCM and judges more directly probe whether drafts preserve staging-critical evidence and decision-relevant consistency under cross-site variation. We additionally report TNM staging outcomes based on the model’s explicit staging predictions; detailed per-stage breakdowns and error analyses are provided in the Supplementary Materials (Section S6). Moreover, a component ablation further shows that TNM-oriented anchors account for most staging gains, while organ-wise retrieval provides a consistent additional improvement in report utility (Supplementary Materials, Section S3).

### TNM staging performance and cross-site robustness

**Fig. 3.**
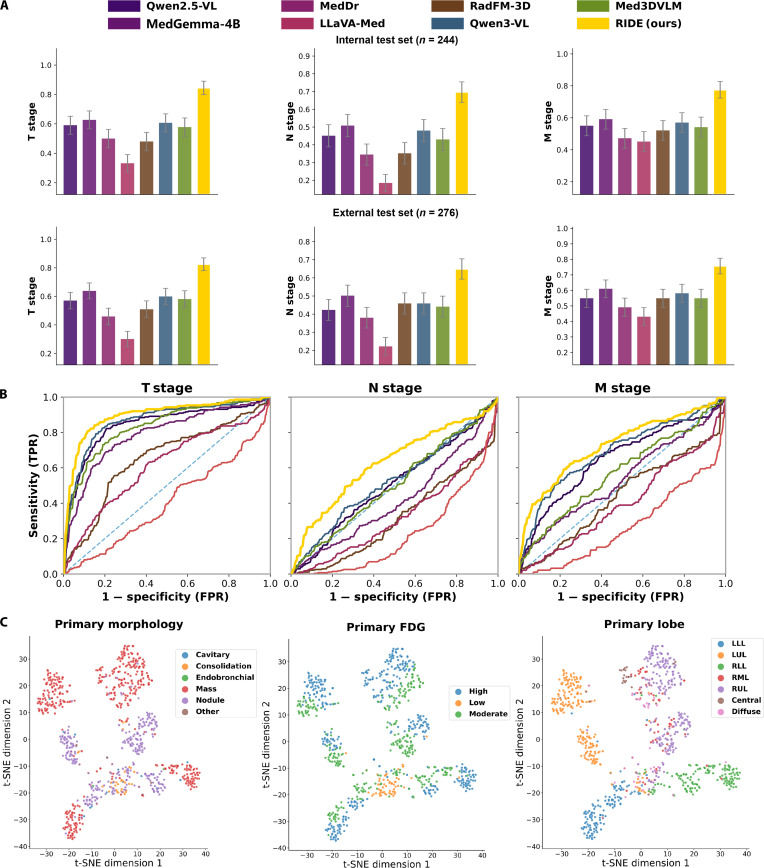
TNM staging performance and representation analysis of the positron emission tomography/computed tomography (PET/CT) encoder. (A) End-to-end TNM staging accuracy evaluated on the internal (*n* = 244) and external (*n* = 276) test sets. Error bars represent 95% bootstrap confidence intervals (CIs), showing RIDE’s higher accuracy with nonoverlapping 95% bootstrap CIs relative to recent 2-dimensional (2D) and 3-dimensional (3D) multimodal baselines. (B) Linear probing performance on frozen PET/CT representations for TNM staging endpoints (T, N, and M stages), reported as receiver operating characteristic (ROC) curves across evaluated methods. (C) t-distributed stochastic neighbor embedding (t-SNE) visualizations of the stage I embedding space, colored by primary morphology (left), primary fluorodeoxyglucose (FDG) uptake level (middle), and primary anatomical lobe (right). The embeddings illustrate pronounced anatomical clustering with partial alignment by morphological and metabolic features.

We explicitly evaluate stage-level accuracy for T, N, and M as high-stakes clinical endpoints (Fig. 3A). RIDE achieves the strongest staging performance across all 3 axes on both internal and external test sets (T/N/M: 85.14/69.62/77.83 internal; 83.29/64.61/76.28 external), corresponding to a stage-averaged accuracy gain of +24.5 points (internal) and +20.4 points (external) over the strongest evaluated baseline (MedGemma-4B). These advantages are observed against both recent 2D generalist models (Qwen2.5-VL, Qwen3-VL, LLaVA-Med, and MedDr) and 3D volumetric baselines (RadFM-3D and Med3DVLM), suggesting that the improvement is not explained by the 3D input format alone.

Against the strongest 2D baseline (Qwen3-VL), RIDE’s external T-stage 95% CI lower bound (78.0%) remains above Qwen3-VL’s upper bound (65.6%) by 12.4 percentage points. A similarly nonoverlapping margin is observed against the strongest 3D baseline (Med3DVLM; 52.3% to 63.9%), indicating that the gain is maintained relative to both 2D and 3D competitors. The N stage remains the most challenging staging axis across all evaluated methods: no baseline exceeds 48.67% (internal) or 45.92% (external), whereas RIDE reaches 69.62% internally and 64.61% externally. This gain is clinically meaningful because the N stage directly influences surgical candidacy: patients staged N0 to N1 may be eligible for curative resection, whereas N2 to N3 disease typically shifts treatment toward definitive chemoradiation. We attribute this improvement to stage I’s explicit, structured prediction of nodal station involvement, which provides discrete, inspectable anchors for downstream synthesis.

M-stage accuracy (77.83% internal, 76.28% external) reflects the challenge of whole-body metastatic surveillance, where distant disease must be detected across multiple organ systems simultaneously. The residual M0 → M1 false positives (FPs; 20.4%) are predominantly driven by severe granulomatous diseases (e.g., active pulmonary tuberculosis) that mimic metastatic patterns on both CT morphology and PET metabolism, representing the physical diagnostic limits of noninvasive imaging rather than a systematic model failure (see the “FP and FN analysis in TNM staging” section for detailed modality ablation).

Cross-site robustness also remains favorable, with only modest internal-to-external accuracy drops (−1.85 for T, −5.01 for N, and −1.55 for M). These drops are generally smaller than those observed for several baselines, supporting reasonable stability under institutional shift. Detailed per-method T/N/M accuracies with 95% bootstrap CIs are provided in Table S8.

### Ablation studies: Volumetric budget and organ-wise retrieval

To disentangle the contribution of volumetric input from that of the model design, we first compared RIDE against three 3D-native medical VLMs under the same full-volume PET/CT input setting. As shown in Tables S13 and S14, RIDE consistently outperforms Med3DVLM, RadFM-3D, and LLaVA-Med across both TNM staging and CRCM metrics on the internal and external cohorts. Because all compared models receive the same complete 3D volumetric input, these results indicate that the observed performance gain cannot be attributed to access to 3D information alone but also reflects the benefit of the TNM-anchored 2-stage design.

We next performed a volumetric information budget study in which the RIDE architecture was held fixed and only the amount of 3D input context was varied. As shown in Table 1, increasing volumetric coverage improves overall performance, especially for the T stage and N stage, whereas the M stage remains comparatively stable across density levels. This pattern suggests that local tumor extent and nodal staging benefit more directly from a broader volumetric context, while distant metastasis detection is less sensitive to the exact slice budget. At the same time, the budget study also shows that volumetric input alone is not sufficient to explain the full performance gap, because the additional advantage of RIDE over other 3D baselines remains even when all models are given complete 3D input. Taken together, the budget study confirms that 3D volumetric context is indeed beneficial, particularly for the T stage and N stage, while the 3D-vs.-3D comparison shows that volumetric input alone does not account for RIDE’s full advantage. Therefore, both factors matter: access to 3D information improves performance, and the TNM-anchored design of RIDE provides an additional gain beyond volumetric input alone.

**Table 1.** Volumetric information budget study. The RIDE architecture is held constant; only input density varies. All 2D baselines (10 slices) are included as anchors. 95% bootstrap CIs in parentheses. Boldface indicates the best performance within each category.

Setting	CRCM-overall ↑	T stage ↑	N stage ↑	M stage ↑
2D baselines (10 slices)				
MedGemma-4B	70.5 (64.8–76.2)	56.6 (50.5–62.7)	44.5 (38.8–50.2)	52.9 (46.8–59.0)
Qwen3-VL	58.9 (52.4–64.1)	60.5 (54.8–65.2)	46.3 (40.2–51.9)	58.7 (52.1–63.6)
Qwen2.5-VL	56.3 (50.8–62.1)	57.2 (51.6–62.7)	42.1 (36.9–48.3)	55.4 (49.1–61.2)
MedDr	59.8 (54.1–65.5)	43.7 (38.1–49.3)	28.5 (23.2–33.8)	40.9 (35.4–46.4)
RIDE variants (architecture fixed, density varied)				
RIDE-2D (10 slices)	58.1 (52.6–64.3)	51.8 (45.2–57.9)	42.6 (36.4–48.1)	74.3 (68.7–79.5)
RIDE-3D (20%)	60.2 (54.9–66.4)	54.1 (49.7–60.3)	40.8 (35.2–46.5)	76.6 (71.3–81.2)
RIDE-3D (40%)	63.7 (57.2–69.8)	61.4 (55.9–66.1)	44.2 (38.5–50.7)	73.5 (68.1–78.9)
RIDE-3D (60%)	80.4 (74.8–84.1)	80.9 (75.2–84.6)	41.5 (36.7–47.2)	75.1 (70.6–80.3)
RIDE-3D (80%)	80.2 (75.9–84.5)	80.1 (75.4–84.3)	62.8 (56.1–67.4)	76.4 (71.5–80.2)
** RIDE-3D (100%)**	**81.8 (76.8–85.9)**	**83.0 (78.0–87.1)**	**65.0 (59.1–70.4)**	**76.0 (70.9–80.7)**

2D, 2-dimensional; CIs, confidence intervals; CRCM, clinical report competency matrix

To directly evaluate the role of retrieval granularity, we compared whole-case retrieval and organ-wise retrieval under identical stage I anchor conditions (Table S1). This comparison shows that retrieval is not uniformly beneficial: when retrieval is performed over the whole case, performance drops relative to anchors-only, with TNM staging decreasing from 81.8/68.2/74.5 to 67.9/55.8/67.0, CRCM from 87.70 to 83.30, and GPT score from 3.72 to 3.17. By contrast, replacing whole-case retrieval with organ-wise retrieval yields the best overall results (83.2/69.0/75.8 TNM, 91.27 CRCM, and 3.89 GPT score). These findings suggest that whole-case retrieval suffers from semantic dilution and cross-organ interference, whereas organ-wise partitioning turns retrieval into a net benefit by keeping evidence anatomically localized and semantically focused.

Whole-case retrieval with anchors does not improve over the anchors-only setting; instead, it reduces both staging accuracy and report quality. This indicates that simply adding more retrieved context is not sufficient and may even be harmful when evidence from multiple organs is mixed into a single long sequence. In contrast, organ-wise retrieval yields consistent gains over anchors-only and substantially outperforms whole-case retrieval under the same anchor condition, supporting the necessity of organ-wise partitioning for stage II retrieval augmentation.

### Multicenter expert and LLM judging: Score composition and calibration

**Fig. 4.**
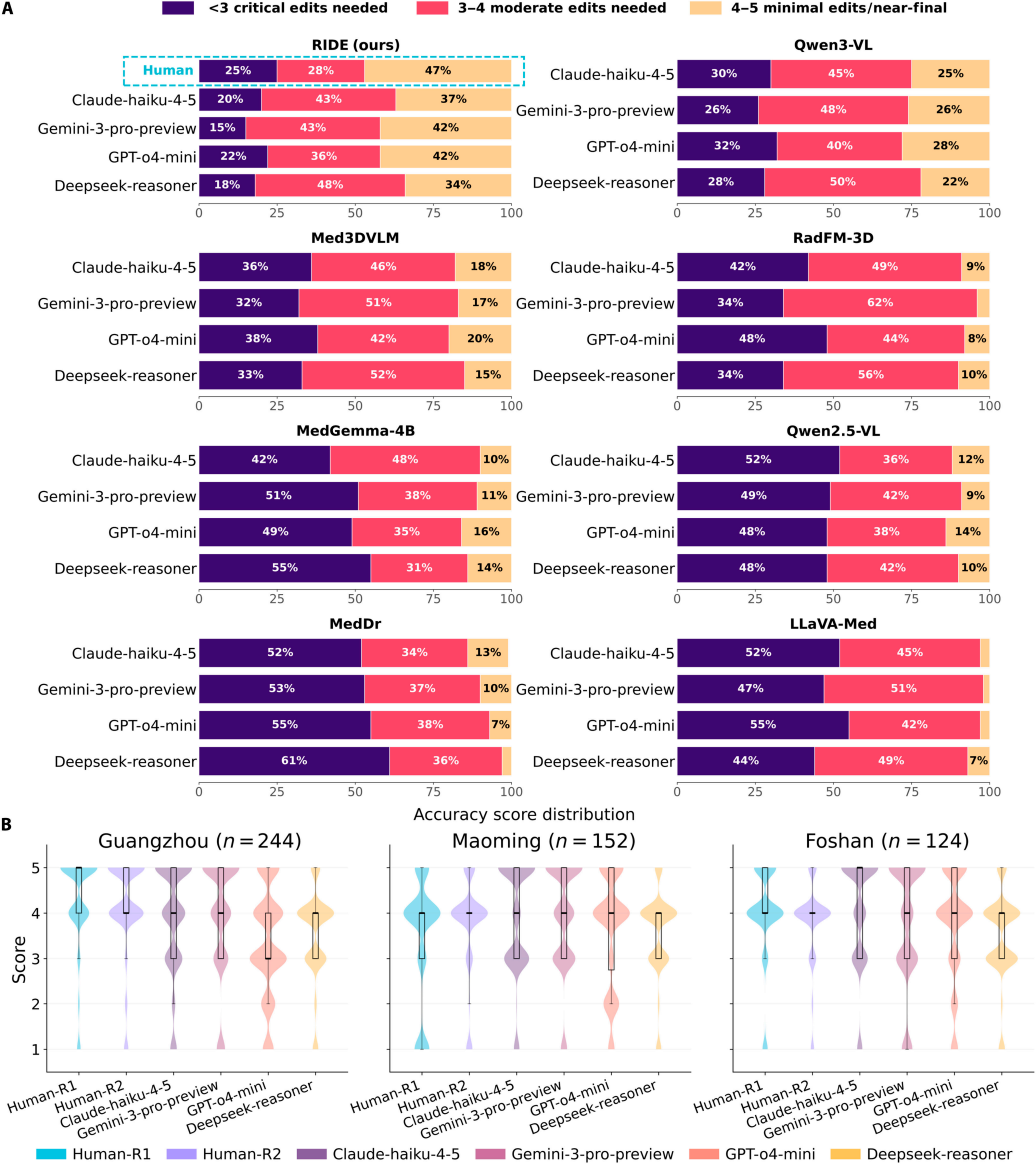
Human and large language model (LLM) judging of report-draft quality under a unified 5-point Likert rubric. (A) Score-band composition of multicenter physician assessment and LLM-based judging. Stacked bars show the percentage of report drafts assigned to 3 postediting tiers: <3 (critical edits needed), 3 to 4 (moderate edits needed), and 4 to 5 (minimal edits/near-final). Two senior nuclear medicine physicians independently scored all RIDE cases (*n* = 520); a senior radiologist with more than 10 years of positron emission tomography/computed tomography (PET/CT) interpretation experience independently blind-scored Qwen3-VL and Med3DVLM on a randomly sampled subset (*n* = 223). In parallel, multiple LLM judges (Claude-haiku-4-5, Gemini-3-pro-preview, GPT-o4-mini, and Deepseek-reasoner) scored all methods using the same rubric to enable calibrated, scalable comparison across systems and centers. (B) Multicenter score distributions by human radiologists and LLM judges. Center-wise violin plots show the distribution of 5-point rubric scores for the proposed system across 3 hospitals (Guangzhou, Maoming, and Foshan), with boxplots summarizing median and interquartile range. Human-R1/Human-R2 denote 2 independent nuclear medicine physicians, while Claude-haiku-4-5, Gemini-3-pro-preview, GPT-o4-mini, and Deepseek-reasoner denote LLM judges scored under the standardized rubric.

Figure 4A summarizes the score-band composition (case-level proportions) under 3 postediting tiers (<3, 3 to 4, and 4 to 5). Human physicians scored all RIDE cases, and their scores show a pronounced right shift: 47% of our drafts fall into the 4-to-5 band, while only 25% require <3-level critical edits. Expert physician scoring remains the primary clinical reference in this study. Direct blinded physician comparison was conducted on a randomly sampled subset (*n* = 223) for RIDE, Qwen3-VL, and Med3DVLM. For the remaining baselines, evaluation was based on automated metrics and LLM-based judges to provide scalable supplementary comparison, but these results are not intended to replace direct physician evaluation. Although LLM judges are systematically more stringent, they preserve the same trend for our drafts, assigning a substantially larger fraction to the top band (34% to 42%) than to the lowest band (15% to 22%). In contrast, baseline VLMs are dominated by lower tiers across judges, typically ~42% to 61% of cases in <3 and only ~7% to 16% in 4 to 5, consistent with frequent staging-relevant omissions or staging-incoherent statements that reduce PET/CT draft utility despite fluent prose. RadFM-3D shifts more cases into the midband (3 to 4) yet still yields a limited near-final fraction (<10%). Overall, the band analysis highlights not only improved average performance but also a markedly better risk profile, with fewer low-score failures and more drafts requiring minimal postediting.

Center-stratified score distributions further support external validity (Fig. 4B). Across the internal center and 2 external centers, both physician raters show highly overlapping distributions, with scores concentrated in the upper range and no systematic degradation on external sites. LLM judges exhibit the expected stricter tendency (a mild downward shift and heavier lower tails) yet preserve the same cross-center consistency, indicating that the model’s report-level fidelity is robust to site-dependent differences in acquisition protocols and reporting habits. We hypothesize that this stability may stem from the staging-first, constraint-aware 2-pass generation, which anchors TNM-critical evidence before refining organ- and lesion-specific descriptors, potentially reducing sensitivity to center-specific stylistic variation.

### Fast impression quality: TNM anchors and representation structure

Because the proposed framework is explicitly anchored by a fast-pass TNM-oriented structured impression, we first quantify how well stage I representations support staging-relevant decision cues. On the multicenter test set, linear probes trained on frozen stage I PET/CT features achieve favorable discrimination for T-, N-, and M-stage endpoints, reported as receiver operating characteristic curves with area under the curve (AUC) (and CIs) across methods (Fig. 3B). Across all 3 axes, the proposed system consistently yields a higher AUC than representative mainstream vision–language baselines, indicating that the fused metabolic–anatomical representation already encodes TNM-critical information in a readily decodable form. This provides quantitative support for using stage I outputs as reliable staging-oriented anchors that guide downstream deliberative synthesis, reducing reliance on unconstrained free-form language generation for high-stakes PET/CT staging claims.

t-SNE visualization further offers an interpretable view of the embedding geometry learned in stage I (Fig. 3B). Embeddings exhibit strong clustering by anatomical location (primary lobe), while morphological features and FDG uptake levels show partial alignment rather than strict partitioning. This organization matches PET/CT reading priors: anatomy provides the dominant spatial scaffold, whereas morphology and uptake refine malignancy assessment and staging evidence along softer, context-dependent boundaries. Together, probing and embedding analyses suggest a coherent mechanism for fast impressions—anatomy-anchored yet metabolically informative representations that are well suited for TNM-oriented anchoring and subsequent report drafting.

### Deliberative synthesis in action: Traceability and case studies

**Fig. 5.**
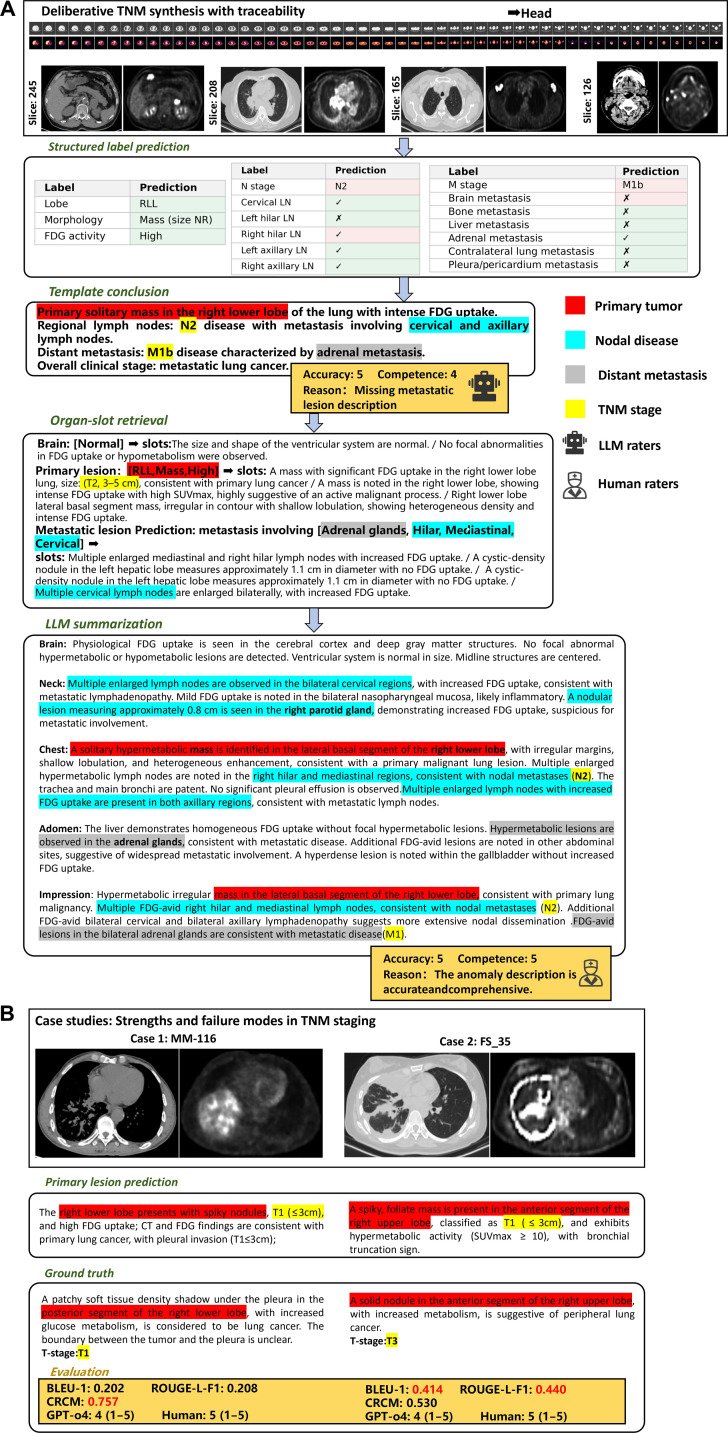
(A) Deliberative synthesis with traceability for a representative positron emission tomography/computed tomography (PET/CT) case. Top: representative PET/CT slices. Middle: stage I structured impression outputs TNM-relevant anchors and a concise template conclusion. Bottom: anchor-conditioned hierarchical organ-wise retrieval returns exemplar organ slots from a routed library (document icons), which are used to prompt a large language model (LLM) to synthesize organ-structured Findings and a TNM-focused Impression. Staging-critical spans in the generated report are highlighted to expose provenance (red, primary tumor; cyan, nodal/metastatic disease; gray, distant metastasis; yellow, T/N/M staging). Score callouts illustrate example judge ratings for intermediate and final drafts (robot, LLM judge; human, clinician). (B) Additional case studies illustrating typical strengths and failure modes. Examples highlight when the generated Impression remains TNM consistent and correctly localizes primary lesions and when errors arise from subtle or sparse metastatic evidence (e.g., omissions or underspecification that can alter staging).

We next illustrate how RIDE converts a fast TNM-oriented impression into a complete whole-body PET/CT draft through deliberative synthesis with reviewable intermediate artifacts (Fig. 5A). In this representative case, stage I first produces a structured impression that explicitly enumerates staging-relevant anchors: primary-lesion attributes (e.g., lobe, morphology, and FDG uptake level) and TNM-critical evidence units for nodal and metastatic disease. These outputs also yield a concise template conclusion, which serves as a starting point for downstream synthesis rather than a final narrative.

Stage II then performs anchor-conditioned, hierarchical organ-wise retrieval. Each stage I anchor routes to a curated organ-slot library, returning exemplar snippets that are (a) organ-specific and (b) directly relevant to the predicted evidence units (document icons in Fig. 5A). This organ-wise routing is designed to reduce cross-organ mixing and improve coverage: it supplies organ-specific phrasing templates aligned with predicted anchors (e.g., involved nodal stations or metastatic organs) and, when routed, provides standard normal/negative templates for organs predicted uninvolved, encouraging whole-body completeness in the final draft.

Finally, an LLM synthesizes organ-structured Findings and a TNM-focused Impression under explicit constraints: it is conditioned on the structured impression and the retrieved organ-wise slots, and it is prompted to reconcile disagreements rather than mechanically copy stage I. This deliberative step can refine staging-critical phrasing when ambiguity exists and incorporate supporting organ context, yielding a report that is simultaneously comprehensive and targeted. Crucially, TNM-relevant spans in the final report are highlighted with provenance cues (red: primary tumor; cyan: nodal/metastatic disease; gray: distant metastasis), enabling a reviewer to rapidly trace each high-stakes statement back to the corresponding stage I anchors and retrieved evidence. This case illustrates how workflow decomposition may improve practical usability for human-in-the-loop PET/CT reporting: it separates what must be correct and checkable (TNM-critical anchors) from how to write a complete narrative (organ-wise synthesis).

Additional case studies further highlight the strengths and failure modes of the proposed system (Fig. 5B). In typical successful cases, the generated Impression is TNM consistent, correctly localizes the primary lesion, and maintains coherent nodal and metastatic statements aligned with the structured impression, while Findings remain organ-wise complete without cross-organ drift. When failures occur, they tend to involve subtle or sparse metastatic evidence, where omission or underspecification can alter staging. Critically, the 2-stage design supports targeted error analysis: discrepancies can be attributed to incorrect fast impression anchors versus insufficient retrieved organ-wise context versus synthesis inconsistencies, enabling focused mitigation strategies without conflating errors with purely linguistic issues.

### Quantitative failure analysis and error attribution

To complement aggregate CRCM and TNM scores, we performed a quantitative failure analysis on the combined validation cohort (*n* = 520). As summarized in Table 2, we report both the number of cases containing at least one error unit and the total number of extracted error units, further decomposed into 4 categories: omission, FP, mislocalization, and misgrading. Among the evaluated methods, the full RIDE model shows the lowest overall error burden (173 cases with ≥1 error unit; 412 total error units). The modality ablations further reveal distinct failure profiles: PET-only markedly increases FPs, whereas CT-only increases omissions, highlighting the complementary roles of metabolic and structural information in reducing different types of report errors. As shown in Table 2, RIDE has the lowest total error count among the evaluated methods. The table also makes the modality effect more interpretable: PET-only chiefly increases FPs, while CT-only chiefly increases omissions. This allows the failure analysis to go beyond aggregate scores and quantify how error composition changes across models.

**Table 2.** Comprehensive report-level error taxonomy based on CRCM unit extraction on the validation set (*n* = 520). The table is structurally divided into 3D models and 2D image/slice-based baselines, ranked approximately by overall performance within their respective groups. While the full RIDE (PET+CT) achieves the lowest total error count among evaluated methods, removing either modality (PET-only or CT-only) severely compromises performance, causing total error units to fall behind top-tier generalist baselines like Qwen3-VL. This underscores the indispensable necessity of synergistic multimodal fusion over purely architectural advantages. Boldface indicates the best performance (lowest error counts) among the evaluated methods.

Model	Cases with ≥1 error unit, *n* (%)	Total error units, *n*	Omission (%)	FP (%)	Mislocalization (%)	Misgrading (%)
3D models and modality ablations						
** RIDE (PET+CT; ours)**	**173 (33.5)**	**412**	**43.2**	**20.4**	**15.3**	**21.1**
Med3DVLM	228 (44.2)	615	37.6	22.7	16.8	22.9
RadFM-3D	235 (45.5)	640	35.7	24.4	17.6	22.3
RIDE (PET-only)	250 (48.4)	685	21.6	42.5	15.2	20.7
RIDE (CT-only)	268 (51.9)	734	51.8	13.4	18.5	16.3
2D models (image/slice-based baselines)						
Qwen3-VL	225 (43.6)	610	33.2	27.5	23.4	15.9
MedGemma-4B	240 (46.5)	655	35.5	24.1	22.5	17.9
Qwen2.5-VL	242 (46.9)	660	34.1	28.8	22.7	14.4
MedDr	296 (57.4)	860	31.5	21.8	29.5	17.2
LLaVA-Med	338 (65.5)	1,100	25.4	36.7	26.8	11.1

CRCM, clinical report competency matrix; 3D, 3-dimensional; 2D, 2-dimensional; PET, positron emission tomography; CT, computed tomography; FP, false positive

We further analyzed the 412 residual error units of RIDE using a multilabel stage attribution framework across Anchor, Retrieval, and Synthesis. Since a single report error may reflect cascading effects, one error unit can be associated with multiple contributing stages. As summarized in Table S9, different error types concentrate in different parts of the pipeline: omissions frequently involve both anchor and retrieval coverage, mislocalization is primarily associated with anchor-stage errors, FPs are more often linked to synthesis, and misgrading commonly involves the retrieval/synthesis interface.

Rather than indicating a single dominant bottleneck, this attribution analysis shows that the residual errors are heterogeneous: localization failures are mostly anchor related, FPs are more often associated with synthesis, and omissions/misgrading often involve retrieval together with other stages. This makes the remaining failure modes more specific and quantitatively interpretable.

### Modality-specific evidence for TNM-critical thoracic anchors

**Fig. 6.**
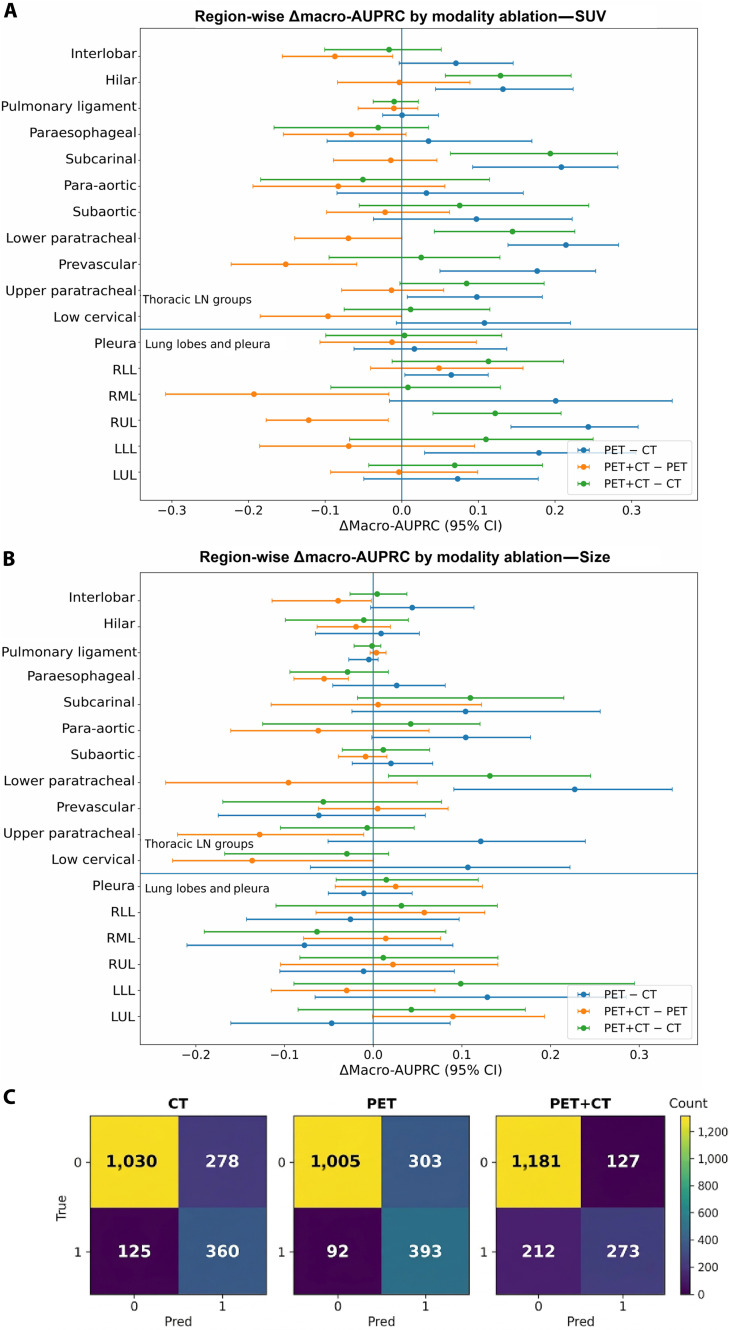
Region-level robustness analysis for TNM-critical thoracic localization. (A) and (B) report region-wise changes in macro–area under the precision–recall curve (macro-AUPRC) (with 95% confidence intervals) under modality ablations for standardized uptake value (SUV)-related and size-related targets, across key thoracic nodal stations and lung/pleural regions. (C) summarizes modality-specific error patterns via confusion matrices for computed tomography (CT)-only, positron emission tomography (PET)-only, and PET+CT settings. Overall, PET+CT provides the most stable performance in lymph-node-rich mediastinal and hilar basins, supporting the need for joint metabolic–anatomical reasoning in TNM-critical staging.

Because stage II drafting is constrained by stage I structured anchors, we probe whether these anchors are supported by genuine dual-modality evidence rather than modality-agnostic language cues. We therefore perform modality ablations and quantify region-wise changes in macro–area under the precision–recall curve (macro-AUPRC) (95% CI) for TNM-relevant thoracic endpoints (Fig. 6A to C). For SUV-related targets, performance is predominantly PET driven: removing PET leads to consistent degradation across nodal stations and lung/pleural regions, whereas adding CT on top of PET yields only modest gains (Fig. 6A), consistent with SUV being a direct quantization of metabolic signal. In contrast, size-related targets show a complementary dependence on CT-derived anatomy, and the effect of removing CT is especially pronounced in mediastinal and hilar lymph-node basins, where small targets, vessel/airway adjacency, and partial-volume effects make PET extent an unreliable proxy for true short-axis size (Fig. 6B). Finally, confusion-matrix patterns further illustrate modality complementarity (Fig. 6C): PET-only predictions tend to incur more spurious positives due to physiologic uptake and spill-in, whereas CT-only predictions more often miss metabolically active disease; joint PET+CT reasoning reduces such modality-specific failure modes by enforcing metabolic–anatomical concordance. Together, these analyses provide explicit mechanistic evidence that our system leverages PET for metabolic staging cues and CT for structural localization, thereby producing more reliable TNM-critical anchors for subsequent retrieval and narrative synthesis. More detailed modality-specific analyses are provided in the Supplementary Materials and in the dedicated modality ablation section (Section S1).

#### FP and FN analysis in TNM staging

For T/N/M staging, modality ablations reveal distinct FP/false negative (FN) trade-offs. PET-only input is associated with substantially elevated FP rates (21.4% for T-stage upstaging, 45.2% for N-stage, and 42.5% for M-stage), whereas CT-only input leads to markedly higher FN rates (30.7% for T-stage downstaging, 41.2% for N-stage, and 48.5% for M-stage). In contrast, the complete PET+CT RIDE framework yields a more balanced error profile across all 3 axes, reducing T-stage upstaging FP to 5.4%, N-stage FP to 24.0%, and M-stage FP to 20.4% while maintaining lower FN rates than CT-only input. For the T stage, the remaining errors are concentrated near adjacent ordinal boundaries rather than large cross-stage jumps, consistent with ambiguity around size thresholds and local invasion assessment. For the M stage, the residual FPs are predominantly associated with severe granulomatous diseases such as active pulmonary tuberculosis, which remain a known diagnostic challenge even under multimodal noninvasive imaging. As shown in Table 3, the complete PET+CT framework consistently shifts the error profile toward a more balanced regime across T/N/M staging rather than improving one axis at the expense of another. In particular, multimodal fusion substantially reduces PET-driven overcalling for the N stage and M stage while avoiding the pronounced undercalling seen with CT-only input.

**Table 3.** T/N/M-stage FP/FN under modality ablations. The full RIDE framework mitigates the high FP rate of PET-only imaging and the high FN rate of CT-only imaging. Boldface outlines the specific error metrics under comparison.

Setting	T stage		N stage		M stage	
	T1–T2 → T3–T4 FP	T3–T4 → T1–T2 FN	N0 → N+ FP	N+ → N0 FN	M0 → M1 FP	M1 → M0 FN
PET-only	21.4	11.1	45.2	14.5	42.5	16.0
CT-only	7.1	30.7	18.4	41.2	12.0	48.5
**RIDE (PET+CT)**	**5.4**	**10.7**	**24.0**	**11.0**	**20.4**	**30.4**

FP, false positive; FN, false negative; PET, positron emission tomography; CT, computed tomography

## Conclusion

This study presents RIDE, a TNM-accountable 2-stage framework for whole-body 3D FDG PET/CT report drafting in lung cancer cohorts. By separating TNM-oriented structured impressions from hierarchical organ-wise exemplar synthesis, RIDE improves report-drafting quality, clinical utility, staging accuracy, and cross-site robustness while preserving explicit, inspectable intermediate outputs for human verification. These findings suggest that workflow-decomposed PET/CT drafting can serve as a practical form of decision support, helping narrow the utility gap between fluent report generation and clinically accountable TNM-relevant conclusions. To the best of our knowledge, this work is the first whole-body 3D FDG PET/CT drafting framework that treats explicit TNM staging as a first-class, image-grounded output. Nevertheless, because the present evaluation is retrospective and limited to lung cancer cohorts, prospective multireader studies and dedicated validation in other malignancies are still needed to establish real-world clinical utility, workflow integration, and broader generalizability.

## Materials and Methods

### Overview: Two-stage TNM-accountable drafting

Our framework follows a 2-stage design (Fig. 1B): a fast pass generates a TNM-oriented structured impression, which serves as an explicit set of staging-relevant anchors, and a second, deliberative pass then performs organ-wise synthesis conditioned on explicit evidence to compose the final report. Concretely, in stage I, we learn a fused 3D PET/CT representation and predict structured TNM-relevant outputs, including primary-lesion attributes and nodal/metastatic evidence units, and translate these structured outputs into a narrative draft by assembling a compact structured-impression scaffold. In stage II, we translate this impression scaffold into a narrative draft by (a) performing hierarchical organ-wise retrieval of exemplar slots from a curated library and (b) prompting an instruction-following LLM as a constrained synthesizer that generates Findings and Impression conditioned only on the scaffold and retrieved slots. This decomposition separates staging-critical anchoring from narrative completion, yielding drafts that are easier to review and less reliant on unconstrained free-form generation in whole-body PET/CT drafting.

### Stage I: Dual-modality representation learning with clinical semantic alignment

Stage I encodes a whole-body PET/CT study into a shared sequence of visual tokens $V\in {\mathbb{R}}^{N\times d}$ using a dual-channel 3D encoder (PET and CT) with lightweight token mixing. From the same token source, we derive pooled study-level embeddings and multiple projected heads that support (a) TNM-critical structured prediction and (b) clinically oriented image–text alignment. This design allows distinct PET/CT semantics (e.g., primary-lesion attributes vs. staging evidence vs. organ-slot patterns) to be emphasized by different heads while remaining grounded in a unified representation:

$$\begin{array}{c} z^{\left(u\right)}=\text{Pool}\left(V\right),\\ z^{\left(k\right)}=W_{k}z^{\left(u\right)},\;k\in K, \end{array}$$ (1)

where $\text{Pool}\left(\cdot \right)$ is permutation-invariant pooling (e.g., mean pooling), $W_{k}$ is a learnable linear projection, and K indexes heads used for structured prediction, schema-level alignment, and organ-slot alignment.

**Multitask structured prediction (TNM-critical supervision).** On top of task-specific embeddings, we train supervised heads for TNM-critical structured endpoints. Primary-lesion descriptors include lobar localization, morphology, multiplicity, lesion size bins, FDG uptake grade, and SUV_max_ bins; staging-related outputs include T stage, N stage, M stage, a coarse stage grouping, and auxiliary multilabel indicators (e.g., organ-level metastasis tags used for organ routing in stage II). The supervised objective combines cross-entropy losses for single-label targets and binary cross-entropy losses for multilabel targets:

$$\begin{array}{c} L_{\sup}=\sum_{m\in K_{\mathrm{CE}}}\lambda_{m}\mathrm{CE}\left(y_{m},\;{\hat{y}}_{m}\right)+\sum_{m\in K_{\mathrm{BCE}}}\lambda_{m}\mathrm{BCE}\left(y_{m},\;{\hat{y}}_{m}\right), \end{array}$$ (2)

where $K_{\mathrm{CE}}$ and $K_{\mathrm{BCE}}$ denote the sets of single-label and multilabel tasks, respectively; $\lambda_{m}$ are task weights; and $\left(y_{m},\;{\hat{y}}_{m}\right)$ are ground-truth labels and predictions, respectively. These outputs constitute the fast-pass structured impression used to build the stage II scaffold.

**Dual-level image–text alignment (InfoNCE/CLIP-style).** To encourage clinically meaningful semantic organization beyond task-head supervision, we apply 2 complementary InfoNCE/CLIP-style alignment losses.

*Schema-level (conclusion) alignment.* We align image embeddings to TNM-oriented conclusion prototypes, i.e., short template-like texts encoding staging-critical semantics and primary-lesion summary patterns. A text encoder maps each prototype to $t^{\left(c\right)}$. For matched pairs $\left(z_{i}^{\left(c\right)},\;t_{i}^{\left(c\right)}\right)$ in a minibatch of size *B*,

$$\begin{array}{c} L_{\text{concl}}=-\frac{1}{B}\sum_{i=1}^{B}\text{log}\frac{\text{exp}\left(\mathrm{sim}\left(z_{i}^{\left(c\right)},\;t_{i}^{\left(c\right)}\right)/\tau \right)}{\sum_{j=1}^{B}\text{exp}\left(\mathrm{sim}\left(z_{i}^{\left(c\right)},\;t_{j}^{\left(c\right)}\right)/\tau \right)}, \end{array}$$ (3)

where $\mathrm{sim}\left(\cdot ,\;\cdot \right)$ is cosine similarity and τ is a temperature.

*Organ-slot alignment.* We further align image embeddings to organ-wise slot prototypes that represent canonical reporting patterns for major anatomic regions in whole-body PET/CT. Let $t^{\left(o\right)}$ be the matched organ-slot text embedding:

$$\begin{array}{c} L_{\text{organ}}=-\frac{1}{B}\sum_{i=1}^{B}\text{log}\frac{\text{exp}\left(\mathrm{sim}\left(z_{i}^{\left(o\right)},\;t_{i}^{\left(o\right)}\right)/\tau \right)}{\sum_{j=1}^{B}\text{exp}\left(\mathrm{sim}\left(z_{i}^{\left(o\right)},\;t_{j}^{\left(o\right)}\right)/\tau \right)}. \end{array}$$ (4)

**Representation alignment objective.** The representation alignment objective is

$$L=L_{\sup}+L_{\text{concl}}+L_{\text{organ}}.$$ (5)

This dual-level alignment encourages the PET/CT representation to organize around staging-relevant conclusions while preserving organ-wise reporting structure.

#### Structured-impression scaffold (schema anchor)

Structured predictions from multitask structured prediction are assembled into a compact structured-impression scaffold that explicitly encodes TNM-relevant elements (primary-lesion attributes, nodal disease evidence, and metastatic evidence) together with PET/CT-specific quantification bins (lesion size and SUV_max_ ranges). The scaffold is (a) directly inspectable for clinician auditing and (b) used as a retrieval key to constrain downstream drafting.

### Stage II: Schema-to-report generation via hierarchical retrieval and evidence-grounded summarization

#### Organ-wise exemplar memory and de-identification

We construct an offline organ-wise exemplar memory from the training-report corpus only. Each training report is segmented into clinically meaningful organ-wise paragraphs (slots), e.g., brain, head-and-neck, thorax/lung, mediastinum/hilum, abdomen/pelvis, and bone. All reports are de-identified prior to indexing by removing protected health information (patient identifiers, accession numbers, dates, and site-specific identifiers). This slotting preserves native report structure and enables retrieval and drafting to follow an organ-consistent narrative.

For clarity, retrieved “evidence slots” are de-identified exemplar snippets from other patients and are used only as linguistic and structural templates (e.g., typical phrasing and organ-wise organization). They are not patient-specific evidence for the current case. All case-specific assertions, including lesions, involved sites, and TNM conclusions, must be constrained by the stage I structured anchors predicted from the input PET/CT.

#### Hierarchical retrieval: Organ routing and within-organ slot selection

Retrieval is performed hierarchically to reduce cross-organ mixing. First, the scaffold triggers organ routing by selecting relevant organ pools using staging cues (e.g., predicted organ-level metastasis indicators). Second, within each routed pool we retrieve top-*K* candidate slots by cosine similarity in a shared embedding space (details in Section S4), and apply lightweight filtering/deduplication to control redundancy and context length. Unless otherwise stated, retrieval uses a fixed similarity floor and a near-duplicate threshold, and the total generator context is capped (e.g., 8,096 tokens) to ensure stable inference; exact hyperparameters are reported in Section S4.

#### Evidence-grounded constrained synthesis

Given the scaffold and retrieved organ-wise slots (grouped by region), we use an instruction-following LLM strictly as a constrained summarizer, not as a free-form diagnostician. The LLM is instructed to (a) treat TNM-critical anchors in the scaffold as hard constraints for all case-specific facts, (b) use retrieved slots only to provide standard phrasing and organ-wise structure (without importing patient-specific findings from exemplars), and (c) perform explicit cross-section consistency checks so that Findings and Impression remain coherent with the same TNM-relevant claims (e.g., nodal status, metastatic status, and primary-lesion localization). Quantitative descriptors (e.g., lesion size or SUV_max_) are generated only from explicit mentions or discrete bins in the scaffold; retrieved slots may provide generic wording templates but must not introduce case-specific numbers.

#### Output template and decoding

The model outputs 2 sections: Findings (organ-wise bullet points in a fixed order: Brain → Head & Neck → Thorax → Abdomen/Pelvis → Other) and Impression (a concise staging-oriented summary derived from the scaffold, with brief recommendations when uncertainty remains). We use Qwen2.5-7B-Instruct for summarization to support local deployment and data-governance constraints. Decoding uses conservative settings (e.g., temperature = 0.3, top-*p* = 0.9, max_tokens = 1,024, and repetition_penalty = 1.1; full details in Section S4).

### Formulation of the CRCM

To formally quantify the clinical utility discussed in the “Evaluation metrics and grading protocol” section, we define the 3 competencies of the CRCM through unit-level matching.

#### Rule-based clinical NLP extraction pipeline

To evaluate structured clinical variables against expert annotations, we used a rule-based clinical NLP pipeline to extract structured information from free-text reports, following a common practice in prior oncology NLP studies [36,37]. Specifically, the pipeline combines 3 established components: (a) Stanford Stanza [38] for syntactic parsing and dependency analysis, (b) the RadLex ontology [39] for anatomical normalization and synonym mapping, and (c) customized NegEx rule sets [40], together with predefined clinical thresholds, to identify negation status and discretize metabolic and malignancy-related variables. Comprehensive details of this extraction algorithm, along with step-by-step matching examples, are provided in Section S12.

#### Expert annotation protocol and discretization

The extracted units are evaluated against an expert-annotated structured dataset (*N* = 831 cases). To standardize the evaluation, we discretize continuous and qualitative findings into categorical levels. Metabolic activity (SUV_max_) is classified into 3 grades: 0 (no obvious uptake; SUV_max_ < 2.5 or missing), 1 (mild to moderate; 2.5 ≤ SUV_max_ ≤ 10), and 2 (marked; SUV_max_ > 10). Similarly, metastasis status is mapped to a 3-level malignancy state: 0 (benign/absent), 1 (indeterminate/suspicious), and 2 (malignant/definite). Based on these standardized units, we calculate the 3 core competencies of the CRCM.

#### Metabolic detection score

The MDS measures whether the generated report correctly identifies regions with abnormal FDG uptake. Let $U_{\mathrm{met}}$ be the set of evaluated metabolic units. We define

$$\mathrm{MDS}=\frac{1}{\left|U_{\mathrm{met}}\right|}\sum_{u\in U_{\mathrm{met}}}I\left[{\hat{y}}_{\mathrm{met}}\left(u\right)=y_{\mathrm{met}}\left(u\right)\right],$$ (6)

where $y_{\mathrm{met}}\left(u\right)$ and ${\hat{y}}_{\mathrm{met}}\left(u\right)$ are the reference and predicted metabolic outcomes for unit *u*, respectively, and $I\left[\cdot \right]$ is the indicator function.

#### Lesion localization score

The LLS quantifies anatomical localization fidelity. Let $U_{\mathrm{loc}}$ be the set of localization units (abnormal findings paired with target anatomical sites):

$$\mathrm{LLS}=\frac{1}{\left|U_{\mathrm{loc}}\right|}\sum_{u\in U_{\mathrm{loc}}}I\left[{\hat{y}}_{\mathrm{loc}}\left(u\right)=y_{\mathrm{loc}}\left(u\right)\right],$$ (7)

where $y_{\mathrm{loc}}\left(u\right)$ and ${\hat{y}}_{\mathrm{loc}}\left(u\right)$ denote the reference and predicted anatomical assignments, respectively.

#### Malignancy classification score

The MCS evaluates whether the report assigns the correct ordered malignancy risk category. Let $U_{\mathrm{mal}}$ be the set of malignancy-labeled units:

$$\mathrm{MCS}=\frac{1}{\left|U_{\mathrm{mal}}\right|}\sum_{u\in U_{\mathrm{mal}}}I\left[{\hat{y}}_{\mathrm{mal}}\left(u\right)=y_{\mathrm{mal}}\left(u\right)\right],$$ (8)

where $y_{\mathrm{mal}}\left(u\right)\in \left\{0,1,2\right\}$ is the reference label (benign/indeterminate/malignant) and ${\hat{y}}_{\mathrm{mal}}\left(u\right)$ is the generated prediction.

#### Agreement with expert reference annotations

To evaluate the consistency of the CRCM extraction pipeline with expert reference annotations, we measured agreement between the automated extraction results and the reference labels across the annotated dataset. The pipeline achieved Cohen’s kappa (κ) values of 0.88 for anatomical localization (LLS), 0.85 for metabolic grading (MDS), and 0.82 for malignancy classification (MCS). These results indicate good agreement with expert reference annotations and support the use of the extraction pipeline for CRCM computation. Representative disagreement cases are further analyzed in the Supplementary Materials.

#### Bootstrap CIs

To quantify uncertainty, we compute case-level bootstrap 95% CIs with 1,000 resamples on the test set for all reported CRCM metrics. The detailed MDS, LLS, and MCS values together with their corresponding 95% CIs are reported in Table S7. Across both internal and external test sets, the resulting confidence ranges remain well separated from the strongest baselines, supporting that the observed CRCM gains are stable under resampling rather than being driven by a small subset of cases. As shown in Fig. 2B, the uncertainty bands widen only modestly on the external cohort, while the ranking of methods remains unchanged. In particular, RIDE preserves the highest lower bound across MDS, LLS, and MCS on both validation sets, which supports the robustness of the CRCM improvements under institutional shift.

### Training and validation protocol

#### Optimization and robustness

Stage I is trained with AdamW [41] and cosine learning-rate scheduling with a short warm-up. We apply mild intensity augmentations (small brightness/contrast perturbations, weak noise, and light blur) designed to preserve anatomical plausibility while improving robustness across centers. Model-selection details (e.g., validation strategy, early stopping criteria, and task-weight settings) and implementation-level hyperparameters are provided in the Supplementary Materials (Section S4).

#### Avoiding information leakage in retrieval

To prevent leakage, the stage II exemplar memory is built exclusively from the training-report corpus and is fixed before evaluation. All retrieval and generation are performed without access to test reports, and de-identification is applied prior to indexing.

### Generalization to other malignancies

Although the present study is validated only on lung cancer cohorts, the framework is modular and may be adaptable to other cancer types because it operates on whole-body PET/CT volumes and report supervision rather than lung-cancer-specific handcrafted rules. However, extending RIDE to other malignancies would require cancer-specific structured targets, exemplar libraries, and dedicated training and validation and is therefore not established by the current study.

### Practical considerations for human-in-the-loop use

#### Computational requirements

Training the complete model requires approximately 29 h on a single NVIDIA H100 graphics processing unit. During inference, processing a full 3D multimodal case takes approximately 28 s (preprocessing, 5 s; forward pass, 18 s; postprocessing, 5 s), with peak memory usage of approximately 64 GB of video random access memory (detailed in Table S11). These measurements suggest that inference time is unlikely to be a major barrier for case-level draft generation, although no prospective workflow timing study was performed.

#### Clinical safety and disagreement handling

A potential risk of 2-stage drafting pipelines is error propagation between stages. In RIDE, the intermediate structured outputs remain inspectable, and cross-stage disagreement can be surfaced rather than silently absorbed into the final narrative. These design choices may support safer physician oversight by making potentially inconsistent cases easier to identify and review against the original PET/CT images. However, formal evaluation of clinical safety and reader interaction will require prospective workflow studies.

#### Workflow integration (human-in-the-loop)

RIDE is intended as an accountable drafting assistant rather than an autonomous diagnostic system. A potential human-in-the-loop usage mode is for physicians to first review the inspectable stage I structured anchors and then review the final narrative draft. When the stage I anchors and stage II narrative are not fully aligned, the system can surface this discrepancy for physician review against the original PET/CT images, with the physician retaining final responsibility for report approval and TNM assignment.

### Limitations

This study has several limitations. First, because full blind physician evaluation for all baselines across the entire 520-case cohort was not feasible, comparative clinical conclusions beyond the sampled physician-evaluated baselines should be interpreted with caution. We partially addressed this by adding blinded physician scoring on a randomly sampled subset for 2 representative strong baselines, while broader cross-method comparisons for the remaining baselines relied on automated assessment. Future multireader, multicase prospective studies will be needed to provide more comprehensive human evaluation across methods.

Second, the present empirical validation is confined to lung cancer cohorts, because all training and evaluation data were derived from patients referred for suspected or confirmed lung cancer. While the framework itself is modular and may be extendable to other malignancies, its performance beyond this setting has not yet been established and will require cancer-specific structured targets, exemplar resources, and dedicated validation.

Third, the current study is retrospective. Accordingly, the observed improvements in report-drafting quality, TNM accountability, and cross-site robustness should be interpreted as evidence supporting the potential clinical utility of the framework, while prospective multireader studies will be important for further characterizing workflow integration, reader interaction, and safety in routine practice.

## Ethical Approval

This retrospective study was conducted in accordance with the Declaration of Helsinki (as revised in 2024) and was approved by the Research Ethics Committee of Guangdong Provincial People’s Hospital (No. S2025-820-01). Individual consent was waived.

## Data Availability

The dataset used and/or analyzed in the current study is available from the corresponding authors on reasonable request. We will release the relevant training code and curated open-source datasets at https://github.com/hugowang010406/PETCT2rep under the Apache 2.0 license. Due to intellectual property and licensing constraints, the proprietary training data used during model pretraining cannot be made publicly available.

## References

[1] Goldstraw P, Crowley J, Chansky K, Giroux DJ, Groome PA, Rami-Porta R, Postmus PE, Rusch V, Sobin L, International Association for the Study of Lung Cancer International Staging Committee. The IASLC Lung Cancer Staging Project: Proposals for the revision of the TNM stage groupings in the forthcoming (seventh) edition of the TNM classification of malignant tumours. J Thorac Oncol. 2007;2(8):706–714. 17762336 10.1097/JTO.0b013e31812f3c1a

[2] Amin MB, Greene FL, Edge SB, Compton CC, Gershenwald JE, Brookland RK, Meyer L, Gress DM, Byrd DR, Winchester DP. The Eighth Edition AJCC Cancer Staging Manual: Continuing to build a bridge from a population-based to a more “personalized” approach to cancer staging. CA Cancer J Clin. 2017;67(2):93–99. 28094848 10.3322/caac.21388

[3] McDonald RJ, Schwartz KM, Eckel LJ, Diehn FE, Hunt CH, Bartholmai BJ, Erickson BJ, Kallmes DF. The effects of changes in utilization and techno logical advancements of cross-sectional imaging on radiologist workload. Acad Radiol. 2015;22(9):1191–1198. 26210525 10.1016/j.acra.2015.05.007

[4] Hosny A, Parmar C, Quackenbush J, Schwartz LH, Aerts HJ. Artificial intelligence in radiology. Nat Rev Cancer. 2018;18(8):500–510. 29777175 10.1038/s41568-018-0016-5 PMC6268174

[5] Zhang X, Meng Z, Lever J, Ho ES. CCD: Mitigating hallucinations in radiology MLLMs via clinical contrastive decoding. arXiv. 2025. 10.48550/arXiv.2509.23379

[6] Chu Y-W, Zhang K, Malon C, Min MR. Reducing hallucinations of medical multimodal large language models with visual retrieval-augmented generation. arXiv. 2025. 10.48550/arXiv.2502.15040

[7] Jiao W, Shang K, Li H, Yan K, Zhang J, Yang G, Guo L, Wan Y, Yang X, Jin D, et al. Vision-language models for automated 3D PET/CT report generation. arXiv. 2025. 10.48550/arXiv.2511.20145

[8] Goswami D, Subedi R, Chakraborty S. MediVLM: A vision language model for radiology report generation from medical images. In: Christodoulopoulos C, Chakraborty T, Rose C, Peng V, editors. *Findings of the Association for Computational Linguistics: EMNLP 2025*. Kerrville, (TX): Association for Computational Linguistics; 2025. p. 10287–10304.

[9] Zhang Y, Wang X, Xu Z, Yu Q, Yuille A, Xu D. When radiology report generation meets knowledge graph. Proc AAAI Conf Artif Intell. 2020;34(07):12910–12917.

[10] Zhang X, Acosta JN, Zhou H-Y, Rajpurkar P. Uncovering knowledge gaps in radiology report generation models through knowledge graphs. arXiv. 2024. 10.48550/arXiv.2408.14397

[11] Fan Y, Yang Z, Liu R, Li M, Chang X. Medical report generation is a multi-label classification problem. arXiv. 2024. 10.48550/arXiv.2409.00250

[12] Jing P, Lee K, Zhang Z, Zhou H, Yuan Z, Gao Z, Zhu L, Papanastasiou G, Fang Y, Yang G. Reason like a radiologist: Chain-of-thought and reinforcement learning for verifiable report generation. arXiv. 2025. 10.48550/arXiv.2504.18453 41412023

[13] Wang C, Zhou W, Ghosh S, Batmanghelich K, Li W. Semantic consistency-based uncertainty quantification for factuality in radiology report generation. In: Chiruzzo L, Ritter A, Wang L, editors. *Findings of the Association for Computational Linguistics: NAACL 2025*. Kerrville, (TX): Association for Computational Linguistics; 2025. p. 1739–1754. 10.18653/v1/2025.findings-naacl.95 PMC12756947 41488130

[14] Li Y, Liang X, Hu Z, Xing EP. Hybrid retrieval-generation reinforced agent for medical image report generation. Adv Neural Inf Proces Syst. 2018;31.

[15] Tao Y, Ma L, Yu J, Zhang H. Memory-based cross-modal semantic alignment network for radiology report generation. IEEE J Biomed Health Inform. 2024;28(7):4145–4156. 38656853 10.1109/JBHI.2024.3393018

[16] Guo K, Zheng S, Huang R, Gao R. Multi-task learning for lung disease classification and report generation via prior graph structure and contrastive learning. IEEE Access. 2023;11:110888–110898.

[17] Zhao G, Feng Q, Chen C, Zhou Z, Yu Y. Diagnose like a radiologist: Hybrid neuro probabilistic reasoning for attribute-based medical image diagnosis. IEEE Trans Pattern Anal Mach Intell. 2021;44(11):7400–7416. 10.1109/TPAMI.2021.3130759 34822325

[18] Choi HS, Song JY, Shin KH, Chang JH, Jang BS. Developing prompts from large language model for extracting clinical information from pathology and ultrasound reports in breast cancer. Radiat Oncol J. 2023;41(3):209. 37793630 10.3857/roj.2023.00633 PMC10556835

[19] Yao Y, Cen X, Gan L, Jiang J, Wang M, Xu Y, Yuan J. Automated esophageal cancer staging from free-text radiology reports: Large language model evaluation study. JMIR Med Inform. 2025;13: Article e75556. 41105871 10.2196/75556 PMC12533932

[20] Truhn D, Eckardt JN, Ferber D, Kather JN. Large language models and multimodal foundation models for precision oncology. NPJ Prec Oncol. 2024;8(1):72. 10.1038/s41698-024-00573-2 PMC10959931 38519519

[21] Hu D, Zhang H, Li S, Wang Y, Wu N, Lu X. Automatic extraction of lung cancer staging information from computed tomography reports: Deep learning approach. JMIR Med Inform. 2021;9(7): Article e27955. 34287213 10.2196/27955 PMC8339987

[22] Artsi Y, Klang E, Collins JD, Glicksberg BS, Nadkarni GN, Korfiatis P, Sorin V. Large language models in radiology reporting—A systematic review of performance, limitations, and clinical implications. medRxiv. 2025. 10.1101/2025.03.18.25324193

[23] Hamamci IE, Er S, Menze B. CT2Rep: Automated radiology report generation for 3D medical imaging. In: Linguraru MG, Dou Q, Feragen A, Giannarou S, Glocker B, Lekadir K, Schnabel JA, editors. *Medical image computing and computer-assisted intervention—MICCAI 2024: 27th international conference, Marrakesh, Morocco, October 6–10, 2024, proceedings, part XII*. Cham (Switzerland): Springer; 2024. p. 476–486.

[24] Chen H, Zhao W, Li Y, Zhong T, Wang Y, Shang Y, Guo L, Han J, Liu T, Liu J, et al. 3D-CT-GPT: Generating 3D radiology reports through integration of large vision-language models. arXiv. 2024. 10.48550/arXiv.2409.19330

[25] Xin Y, Ates GC, Gong K, Shao W. Med3DVLM: An efficient vision-language model for 3D medical image analysis. arXiv. 2025. 10.48550/arXiv.2503.20047 40889320

[26] Oh Y, Seifert R, Cao Y, Clement C, Ferdinandus J, Lapa C, Liebich A, Amon M, Enke J, Song S, et al. Developing a PET/CT foundation model for cross-modal anatomical and functional imaging. arXiv. 2025. 10.48550/arXiv.2503.02824

[27] Sellergren A, Kazemzadeh S, Jaroensri T, Kiraly A, Traverse M, Kohlberger T, Xu S, Jamil F, Hughes C, Lau C, et al. MedGemma technical report. arXiv. 2025. 10.48550/arXiv.2507.05201

[28] He S, Nie Y, Chen Z, Cai Z, Wang H, Yang S, Chen H. MedDr: Diagnosis-guided bootstrapping for large-scale medical vision-language learning. arXiv. 2024. https://arxiv.org/abs/2404.15127v1

[29] Wu C, Zhang X, Zhang Y, Hui H, Wang Y, Xie W. Towards generalist foundation model for radiology by leveraging web-scale 2D&3D medical data. Nat Commun. 2025;16(1):7866. 40849424 10.1038/s41467-025-62385-7 PMC12375113

[30] Bai J, Bai S, Yang S, Wang S, Tan S, Wang P, Lin J, Zhou C, Zhou J. Qwen-VL: A frontier large vision-language model with versatile abilities. arXiv. 2023. https://arxiv.org/abs/2308.12966v1

[31] Li C, Wong C, Zhang S, Usuyama N, Liu H, Yang J, Naumann T, Poon H, Gao J. LLaVA-Med: Training a large language-and-vision assistant for biomedicine in one day. Adv Neural Inf Proces Syst. 2023;36:28541–28564.

[32] Suzuki K. Claude 3.5 Sonnet indicated improved TNM classification on radiology report of pancreatic cancer. Jpn J Radiol. 2025;43(1):56–57. 39404923 10.1007/s11604-024-01681-6

[33] Team G, Georgiev P, Lei VI, Burnell R, Bai L, Gulati A, Tanzer G, Vincent D, Pan Z, Wang S, et al. Gemini 1.5: Unlocking multimodal understanding across millions of tokens of context. arXiv. 2024. 10.48550/arXiv.2403.05530

[34] Jaech A, Kalai A, Lerer A, Richardson A, El-Kishky A, Low A, Helyar A, Madry A, Beutel A, Carney A, et al. OpenAI o1 system card. arXiv. 2024. 10.48550/arXiv.2412.16720

[35] DeepSeek-AI, Liu A, Feng B, Xue B, Wang B, Wu B, Lu C, Zhao C, Deng C, Zhang C, et al. Deepseek-V3 technical report. arXiv. 2024. 10.48550/arXiv.2412.19437

[36] Kehl KL, Xu W, Gusev A, Bakouny Z, Choueiri TK, Riaz IB, Elmarakeby H, Van Allen EM, Schrag D. Artificial intelligence-aided clinical annotation of a large multi-cancer genomic dataset. Nat Commun. 2021;12(1):7304. 34911934 10.1038/s41467-021-27358-6 PMC8674229

[37] Kehl KL, Elmarakeby H, Nishino M, Van Allen EM, Lepisto EM, Hassett MJ, Johnson BE, Schrag D. Assessment of deep natural language processing in ascertaining oncologic outcomes from radiology reports. JAMA Oncol. 2019;5(10):1421–1429. 31343664 10.1001/jamaoncol.2019.1800 PMC6659158

[38] Qi P, Zhang Y, Zhang Y, Bolton J, Manning CD. Stanza: A Python natural language processing toolkit for many human languages. In: Celikyilmaz A, Wen T-H, editors. *Proceedings of the 58th annual meeting of the Association for Computational Linguistics: System demonstrations*. Kerrville, (TX): Association for Computational Linguistics; 2020. p. 101–108.

[39] Langlotz CP. RadLex: A new method for indexing online educational materials. Radiographics. 2006;26(6):1595–1597. 17102038 10.1148/rg.266065168

[40] Chapman WW, Bridewell W, Hanbury P, Cooper GF, Buchanan BG. A simple algorithm for identifying negated findings and diseases in discharge summaries. J Biomed Inform. 2001;34(5):301–310. 12123149 10.1006/jbin.2001.1029

[41] Kingma DP, Ba J. Adam: A method for stochastic optimization. arXiv. 2014. 10.48550/arXiv.1412.6980

