# Supplementary material for: TNM-Accountable Whole-Body 3-Dimensional Fluorodeoxyglucose Positron Emission Tomography/Computed Tomography Report Drafting in Lung Cancer Cohorts via Structured Impressions and Organ-wise Exemplar Synthesis
Source: Research (Wash D C). 2026 Jul 21;9:1343. doi: 10.34133/research.1343 (PMC13385537; doi:10.34133/research.1343)
Supplement: Supplementary 1 — Supplementary Text Figs. S1 to S7 Tables S1 to S14 [file research.1343.f1.zip › Supplementary.pdf]

Supplementary Materials for TNM-Accountable Whole-Body 3D FDG PET/CT Report Drafting in Lung Cancer Cohorts via Structured Impressions and Organ-Wise Exemplar Synthesis

Hang Wang[1,2,3†], Xingyu Xie[1,2†], Entao Liu[4†], Yunlong Gao[1,2], Xiaorui Wu[1,2], Shan Cong[3], Jidong Han[1,2], Na Zhang[1,2], Hairong Zheng[1,2], Dong Liang[1,2], Xiaohui Yao[3*], Lei Jiang[4*], and Zhanli Hu[1,2*]

[1]The Research Center for Medical AI, Shenzhen Institute of Advanced Technology, Chinese Academy of Sciences, Shenzhen 518055, Shenzhen, China

[2]The Key Laboratory of Biomedical Imaging Science and System, Chinese Academy of Sciences, State Key Laboratory of Biomedical Imaging Science and System, Shenzhen, China.

[3]College of Intelligent Systems Science and Engineering, Harbin Engineering University, Harbin 150001, Heilongjiang, Harbin, China

[4]PET Center, Department of Nuclear Medicine, Guangdong Provincial People's Hospital (Guangdong Academy of Medical Sciences), Southern Medical University, Guangzhou, China

[*]Address correspondence to: zl.hu@siat.ac.cn (Z. Hu), leijiang1031@163.com (L. Jiang), xiaohui.yao@hrbeu.edu.cn (X. Yao).

[†]These authors contributed equally to this work.

Contents

S1 Additional results: modality ablation confusion matrices **3**

S2 Physician–LLM score calibration **6**

S3 System ablation: contributions of TNM anchors and organ-wise retrieval **6**

S4 Implementation details **8**
- S4.1 Evidence library and de-identification 8
- S4.2 Hierarchical retrieval details 8
- S4.3 Evidence-grounded summarization protocol (non-verbatim) 10
- S4.4 LLM selection rationale 10
- S4.5 Checklists used in the 5-point physician rubric. 11

S5 Validation of LLM-based Evaluation via Human Radiologist Audit **11**

S6 TNM Confusion Matrices **14**

S7 Bootstrap confidence intervals **15**

S8 Stage Attribution of Errors **16**

S9 Synthesis Inconsistency Failure Modes **17**

S10 Inference Time and Resource Consumption **17**

S11 Standard Linguistic Evaluation Metrics Formulation **18**

S12 Implementation Detail and work example **18**
- S12.1 NLP Pipeline and Terminology Mapping 19
- S12.2 Step-by-Step Metric Computation 19
- S12.3 Limitations of the CRCM NLP Extraction Tool: Failure Case Analysis 20
- S12.4 Multi-Label Root Cause Analysis 21
- S12.5 Metric Reliability Confirmation 22

S13 Comparison against 3D-native baselines **22**

S1 Additional results: modality ablation confusion matrices

We report row-normalized confusion matrices under modality ablation (CT-only, PET-only, PET+CT) for three structured endpoints used in Stage I: META (binary), SIZE-bin (5-way), and SUV-bin (3-way). For each endpoint, results are shown across three thoracic region sets (Thorax overall, Lung lobes & pleura, Thoracic LN groups). Rows denote ground-truth classes and columns denote predictions; diagonal mass indicates per-class recall.

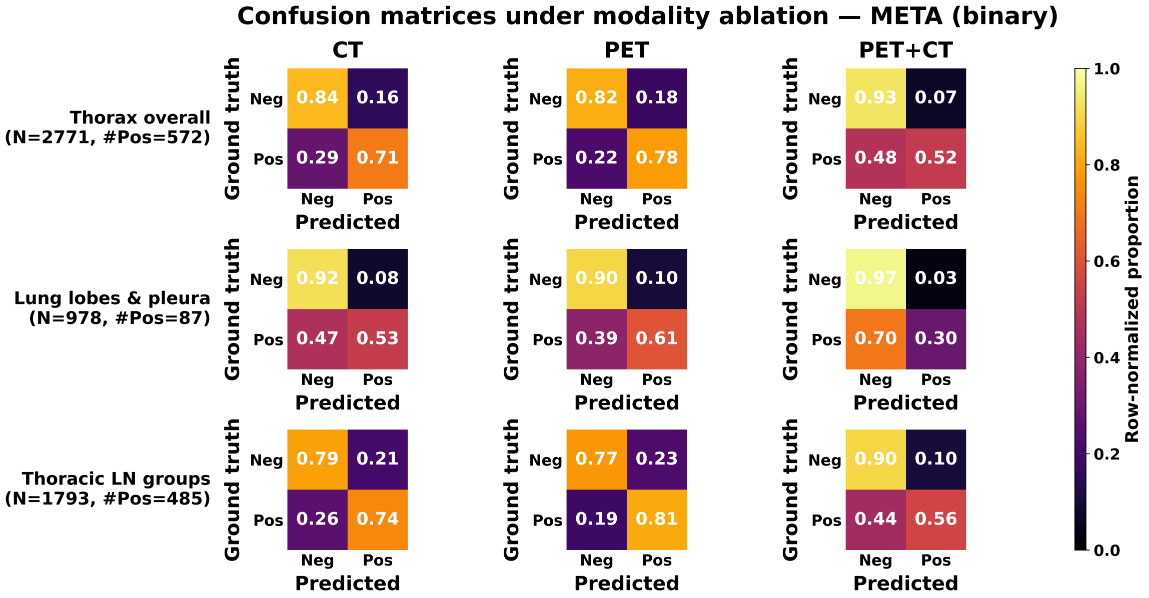

Figure S1: **META (binary) confusion matrices under modality ablation.** Row-normalized confusion matrices for CT-only, PET-only, and PET+CT across three thoracic region sets. N (and #Pos) are shown for each region set.

META (binary). PET-only yields higher sensitivity for META-positive cases (higher Pos→Pos), consistent with metabolic signal being informative for metastatic involvement. In contrast, PET+CT achieves the highest Neg→Neg proportions but shows increased Pos→Neg errors in some subsets, suggesting a conservative bias toward negative predictions under the current fusion setting. Lung lobes & pleura remains the most challenging subset, likely reflecting lower positive prevalence and greater heterogeneity.

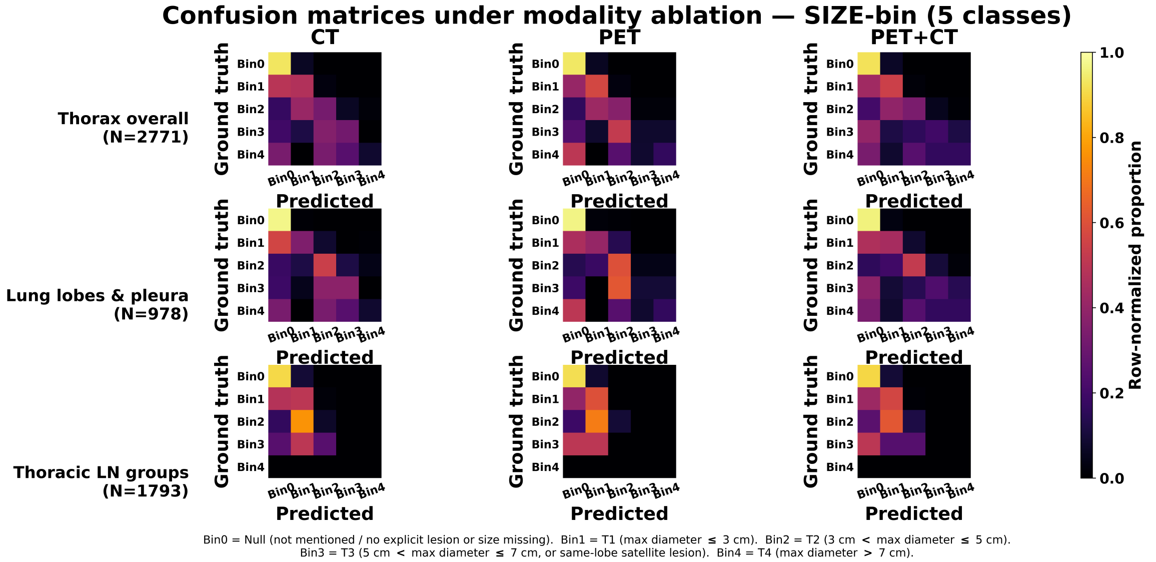

Figure S2: **SIZE-bin (5-way) confusion matrices under modality ablation.** Row-normalized confusion matrices across thoracic region sets for CT-only, PET-only, and PET+CT. Bins follow TNM diameter thresholds: Bin0 (missing/unspecified), Bin1 ($\leq$3 cm), Bin2 (3–5 cm), Bin3 (5–7 cm or same-lobe satellite), Bin4 ($>$7 cm).

SIZE-bin (5-way). Errors are dominated by adjacent-bin confusions, consistent with threshold-based discretization of a continuous diameter variable. CT-only shows sharper separation among intermediate-to-large bins, aligning with CT providing direct morphologic cues for lesion extent. PET-only exhibits greater bin mixing, while PET+CT partially mitigates this mixing but does not eliminate boundary ambiguity. Misassignments involving Bin0 mainly reflect missing or non-explicit size mentions rather than true disagreement on measured diameter.

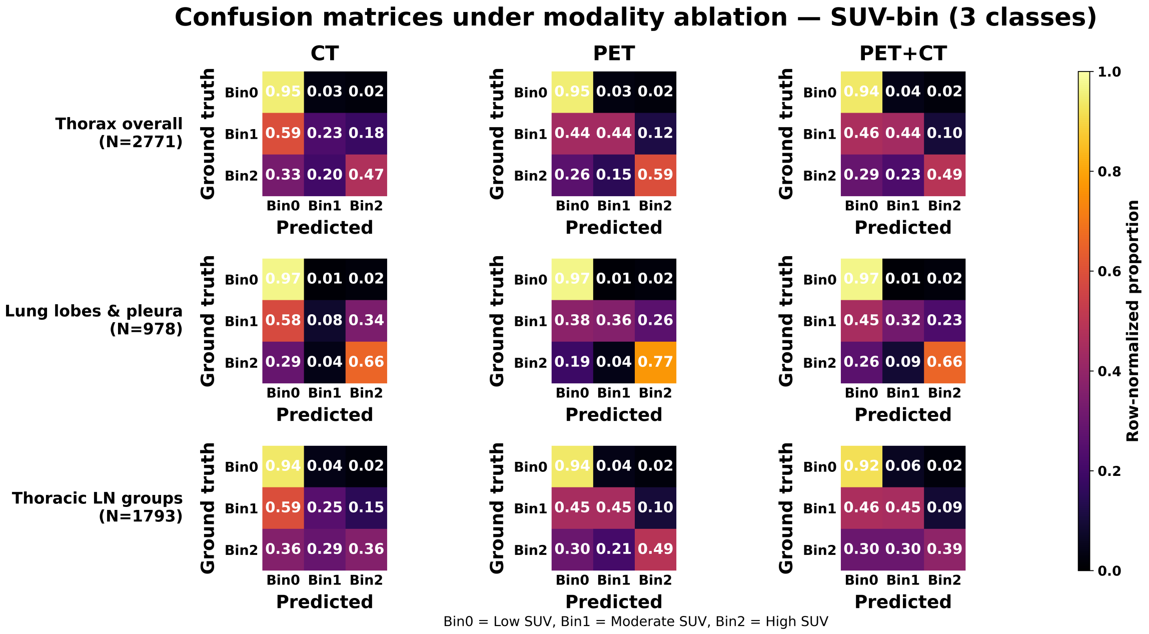

Figure S3: **SUV-bin (3-way) confusion matrices under modality ablation.** Row-normalized confusion matrices across thoracic region sets for CT-only, PET-only, and PET+CT. Bins: Bin0 (low), Bin1 (moderate), Bin2 (high).

SUV-bin (3-way). PET predominantly governs separability for moderate-to-high uptake: relative to CT-only, PET-only substantially reduces Bin1→Bin0 leakage and improves Bin2 recall across region sets. PET+CT maintains strong specificity for low SUV (high Bin0 diagonal) but does not uniformly improve Bin2 recall beyond PET-only, again indicating a conservative bias under the current fusion configuration. These patterns suggest that bin-aware calibration or cost-sensitive training may be beneficial when sensitivity to higher uptake is clinically prioritized.

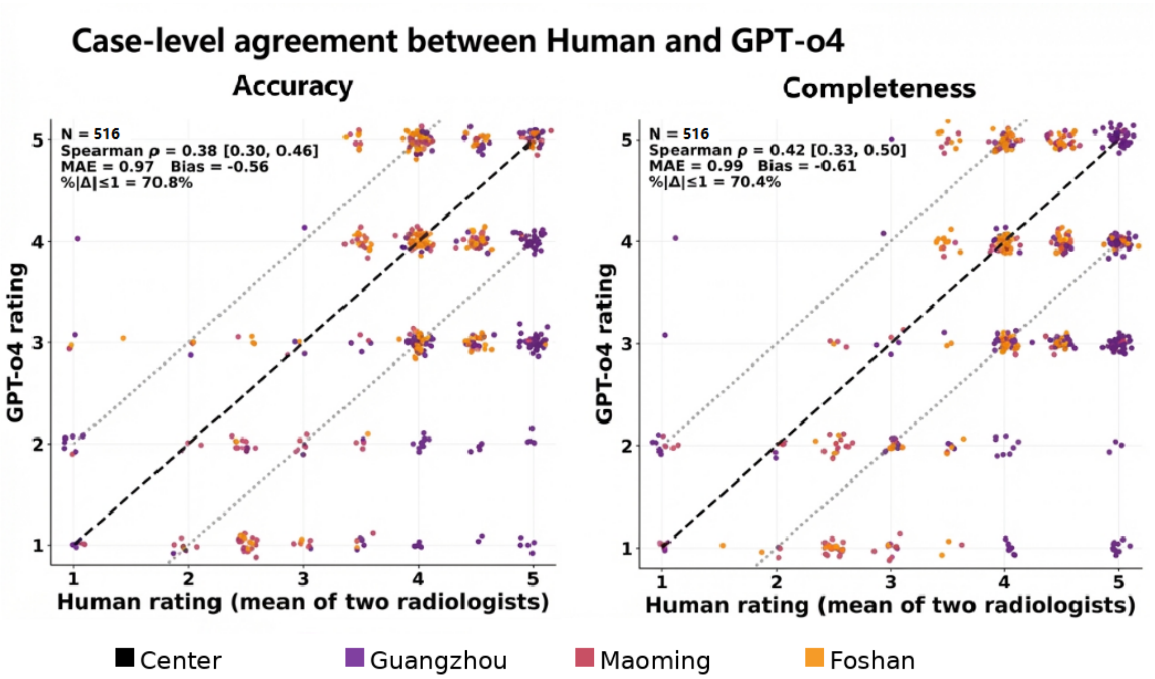

Figure S4: **Case-level calibration between physicians and GPT-o4.** Case-level agreement between the mean physician rating (two nuclear medicine physicians) and GPT-o4 for *Accuracy* and *Completeness* on RIDE-generated reports ($N = 520$). The plots summarize rank agreement (Spearman's ρ with bootstrap confidence intervals) and systematic rating offset (bias), with approximately 70% of cases within ± 1 Likert point.

S2 Physician–LLM score calibration

We assess the case-level agreement between physician readers and a representative top LLM judge (GPT-o4) under the same 5-point rubric (Fig. S4). Across all RIDE cases from three centers ($n = 520$), GPT-o4 exhibits a clear monotonic association with the mean physician rating for both Accuracy ($\rho = 0.38$ [0.30, 0.46]) and Completeness ($\rho = 0.42$ [0.33, 0.50]). Despite this rank-level agreement, GPT-o4 is systematically more stringent, with a negative mean bias of approximately -0.6 Likert points (MAE ≈ 1.0) and about 70% of cases falling within ± 1 point of the physician score. This calibration pattern indicates that LLM judging can reliably track physician preferences at the case level while applying a consistently harsher standard, often penalizing under-specified lesion descriptors or missing low-salience findings.

S3 System ablation: contributions of TNM anchors and organ-wise retrieval

Table S1: **Component ablation of RIDE.** We quantify the contributions of (i) TNM-oriented anchors (Stage I structured impressions) and (ii) retrieval augmentation in Stage II. TNM-T/N/M are staging accuracies (%), CRCM Avg. is the average of the clinically grounded competency metrics, and GPT score is the LLM-judge rating.

Setting	TNM-T	TNM-N	TNM-M	CRCM Avg.	GPT score
End-to-end LLM (no anchors, no retrieval)	29.4	19.8	25.1	19.5	1.78
Whole-case RAG (no anchors)	38.6	28.5	34.2	24.5	2.10
Whole-case RAG (anchors)	67.9	55.8	67.0	83.30	3.17
Anchors-only (no retrieval)	81.8	68.2	74.5	87.70	3.72
**RIDE (anchors + organ-wise RAG)**	**83.2**	**69.0**	**75.8**	**91.27**	**3.89**

Retrieval without anchors is not sufficient for PET/CT staging accountability. Whole-case RAG (no anchors) provides only modest gains over the end-to-end baseline in TNM accuracy and CRCM (e.g., CRCM: 19.5 → 24.5; GPT: 1.78 → 2.10), suggesting that case-level exemplar conditioning alone does not reliably recover TNM-critical evidence or decision consistency.

Adding organ-wise retrieval on top of anchors provides a consistent additional benefit, especially on clinical utility and judge ratings (CRCM: 87.70 → 91.27; GPT: 3.72 → 3.89), with modest gains in TNM accuracies. This pattern is expected: once Stage I anchors already encode most staging-critical cues, retrieval primarily improves organ-wise completeness and evidence-conditioned phrasing rather than changing the core staging decisions.

S4 Implementation details

S4.1 Evidence library and de-identification

Evidence source and split policy. The evidence library is constructed exclusively from the training split. Reports from the test and external splits are used only as queries and are never indexed, to minimize the risk of near-verbatim leakage and to ensure that retrieval reflects generalizable patterns rather than memorization.

De-identification. All reports are de-identified prior to indexing. We remove protected health information (PHI), including (but not limited to) patient names, direct identifiers (e.g., MRN/accession numbers in report text), calendar dates/timestamps appearing in the text, institution/center identifiers, scanner/exam IDs, and other direct identifiers. Acquisition timestamps are used only for split-safety filtering ($\pm$24 h) and are not included in the retrieved slot text or embeddings. We additionally remove free-text signatures and rare identifiers if present.

Split-safety keys. We retain hashed case/patient keys solely for retrieval filtering; these keys are never stored in evidence text nor exposed to the LLM.

Organ-wise slotting. Each report is segmented into organ-wise paragraphs ("evidence slots") following the original report's anatomical organization. Slots correspond to clinically meaningful organ sections (e.g., brain, head-and-neck, thorax/lung, mediastinum/hilum, abdomen/pelvis, bone/other). This choice preserves the narrative structure commonly used in whole-body PET/CT reporting and enables hierarchical retrieval and generation to align with organ-by-organ reporting conventions. To control context length and reduce topic drift, each slot is capped at 400 tokens.

Example of slot segmentation (illustrative). A typical report is split into the following slot sequence (when present):

- `[BRAIN]` …
- `[HEAD & NECK]` …
- `[THORAX]` … (primary lesion and thoracic nodal groups)
- `[ABDOMEN/PELVIS]` …
- `[BONE/OTHER]` …

Slots are treated as independent retrieval units and later concatenated in the same anatomical order during report drafting.

S4.2 Hierarchical retrieval details

Organ pools (pre-bucketed indices). We build separate vector indices ("pools") per organ group to support structure-aware retrieval:

- Brain pool
- Head & neck pool

- Chest/Thorax pool (including lung and thoracic nodal descriptions such as hilum/mediastinum)
- Abdomen/Pelvis pool
- Bone/Other pool

Embedding model and similarity. All evidence slots are embedded using `Qwen/Qwen3-Embedding-8B` with embedding dimension 768. Retrieval is performed by cosine similarity in the embedding space.

Hierarchical retrieval procedure. Retrieval proceeds in two steps:

1. **Schema-driven organ routing.** The Stage 1 schema anchor contains organ-level metastatic predictions (multi-hot). If a target organ is predicted positive (e.g., brain-positive), retrieval is routed to the corresponding organ pool. For each routed organ, we optionally compose an organ-specific descriptor based on predicted attributes (e.g., SUV-bin labels) to guide retrieval within that pool. Routing prioritizes predicted positive organs; chest evidence is always retrieved given its staging relevance in our cohort.

2. **Within-pool retrieval with per-organ budgets.** Within each routed organ pool, we retrieve evidence slots subject to fixed per-organ budgets (reflecting typical report length distribution):

   - Brain / Head & neck / Bone: up to 2 slots each
   - Chest/Thorax: up to 5 slots
   - Abdomen/Pelvis: up to 3 slots

   Retrieved slots with cosine similarity below 0.3 are discarded (`slot_presence_threshold` = 0.3).

Slot-to-slot deduplication. To reduce redundancy, we apply slot-to-slot deduplication within each organ block. If a newly selected slot has cosine similarity above 0.9 to any already selected slot in the same organ block, it is treated as a near-duplicate and removed (`rag_similarity_threshold` = 0.9). Thresholds (slot presence 0.3; deduplication 0.9) were selected on a held-out validation subset to balance evidence coverage and redundancy.

Cross-split safety filtering. Although the index is constructed from training data only, we further exclude any candidate slots that share the same `case_id` or `patient_id` with the query case, or originate from the same center within a ± 24 h acquisition window, to prevent overlap in repeated examinations and reduce the chance of near-duplicate evidence being retrieved.

Context assembly. All retained slots are concatenated into organ-wise evidence blocks with organ headers (e.g., `[THORAX]`) and ordered as Brain $\rightarrow$ Head & Neck $\rightarrow$ Thorax $\rightarrow$ Abdomen/Pelvis $\rightarrow$ Bone/Other. The total context passed to the generator is capped at 8096 tokens.

S4.3 Evidence-grounded summarization protocol (non-verbatim)

Input components. The summarizer receives (i) a schema anchor containing TNM-relevant cues and routing hints, and (ii) a set of organ-wise evidence blocks retrieved from the training-only evidence library. Each evidence block is prefixed with an organ header (e.g., `[THORAX]`, `[BRAIN]`) and contains up to a fixed number of evidence slots (brain/neck/bone ≤ 2; chest ≤ 5; abdomen/pelvis ≤ 3), with total context capped at 8096 tokens.

Constraint rules (anchor-first; exemplar-as-template):

1. All case-specific lesions, metastatic sites, and TNM conclusions must be supported by the schema anchor predicted from the input PET/CT. Retrieved exemplar blocks are used only for language/structure templates and must not be treated as patient-specific evidence.

2. Quantitative statements (e.g., size or SUV) may only be included when present in the schema anchor (as explicit mentions or discrete bins). Exemplar blocks may provide generic phrasing but must not introduce case-specific numbers.

3. The output must follow the fixed report template below. When the schema anchor is uncertain or incomplete, the model must express uncertainty and defer to clinician review rather than speculate.

Fixed report template (structure).

- **Findings:**
  - Brain: bullet points (if routed)
  - Head & Neck: bullet points (if routed)
  - Thorax: bullet points (including primary lesion and thoracic nodal groups)
  - Abdomen/Pelvis: bullet points (if routed)
  - Bone/Other: bullet points (if routed)
- **Impression:** staging-oriented summary derived from the schema anchor.

Note on prompt disclosure. To support reproducibility while reducing the risk of prompt-specific overfitting or misuse, we do not release the verbatim system prompt. The protocol above captures the full constraint logic and output structure used in all experiments.

S4.4 LLM selection rationale

We use `Qwen/Qwen2.5-7B-Instruct` for evidence-grounded summarization because it can be deployed locally, which supports reproducibility, latency constraints, and data-governance requirements common in clinical environments. Our choice prioritizes (i) adherence to evidence-only constraints, (ii) compliance with the fixed output template, and (iii) stability under low-temperature decoding. We do not rely on the LLM for diagnostic reasoning; instead, it acts as a controlled paraphraser that improves readability while preserving schema- and evidence-supported content.

S4.5 Checklists used in the 5-point physician rubric.

Table S2: Checklists used in the 5-point physician rubric.

Category	Items
Anatomical regions (21)	*Lower neck supraclavicular and sternal notch; Paratracheal; Vascular and retrotracheal; Lower paratracheal; Subaortic; Para-aortic; Subcarinal; Paraesophageal; Pulmonary ligament; Hilar; Interlobar and peripheral; Left upper lobe; Left lower lobe; Right upper lobe; Right middle lobe; Right lower lobe; Kidney; Liver; Adrenal gland; Pleura; Sternum*

S5 Validation of LLM-based Evaluation via Human Radiologist Audit

To provide an additional human reference for comparative evaluation, we conducted a blinded physician assessment on a randomly sampled subset of 223 cases drawn from the multi-center external test sets (Guangzhou, Maoming, and Foshan). A senior radiologist with more than 10 years of PET/CT interpretation experience independently scored the reports generated by RIDE, Qwen3-VL, and Med3DVLM. The evaluation followed the same 5-point Likert scale used in our automated assessment framework, focusing on report accuracy and completeness.

As summarized in Table S3, RIDE achieved the highest mean human evaluation score (4.28 ± 0.36), compared with Qwen3-VL (3.48 ± 0.40) and Med3DVLM (3.15 ± 0.52). Pairwise comparisons using paired t-tests showed that the differences between RIDE and each baseline were statistically significant ($p < 0.001$). Figure S5 further illustrates the score distributions across the three methods, with RIDE showing generally higher ratings and a more concentrated distribution in the upper score range.

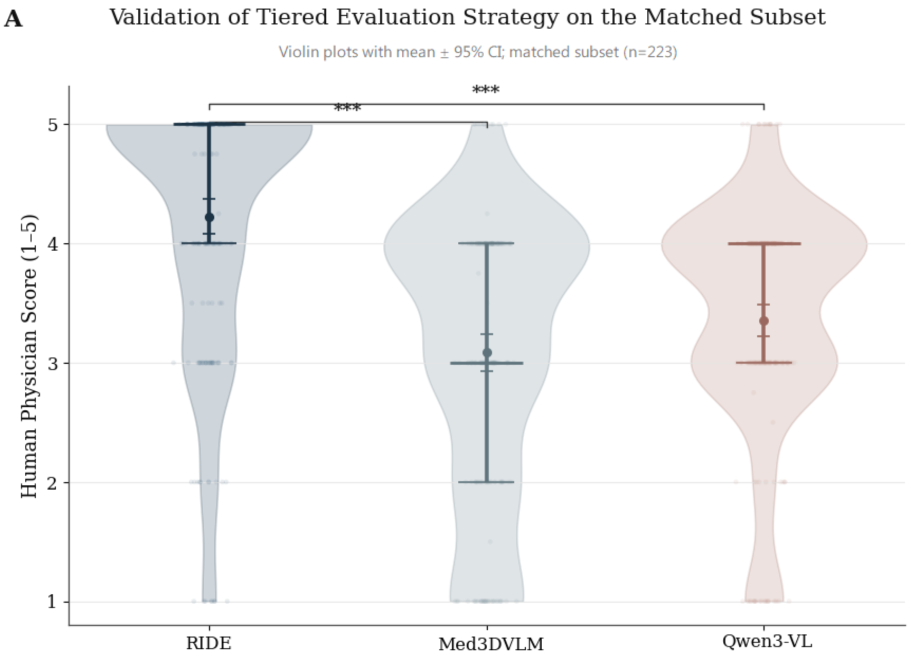

Figure S5: **Distribution of blinded human evaluation scores (n = 223).** The violin plots illustrate the density of scores for RIDE and two SOTA baselines, overlaid with individual data points and mean ± SD error bars. RIDE demonstrates a significantly higher median and tighter distribution compared to Qwen3-VL and Med3DVLM (*** indicates $p < 0.001$).

Figure S5 shows the distribution of physician-assigned scores across the three models on the sampled subset. The figure complements the summary statistics in Table S3 by illustrating the case-level score distributions for each method. The mean score for RIDE (4.28 ± 0.36) was higher than those of Qwen3-VL (3.48 ± 0.40) and Med3DVLM (3.15 ± 0.52), with pairwise differences remaining statistically significant ($p < 0.001$).

Table S3: Quantitative results of blinded human evaluation ($n = 223$).

**Model**	**Mean Score**	**Std. Dev. (SD)**	**p-value (vs. RIDE)**
**RIDE (Ours)**	**4.28**	**0.36**	-
Qwen3-VL	3.48	0.40	< 0.001
Med3DVLM	3.15	0.52	< 0.001

Table S3 provides the precise numerical summary of the human audit. Beyond the mean scores, we analyzed the Spearman rank correlation (ρ) to calibrate our automated LLM judges.

- **Table Description:** The table lists the mean human scores, standard deviations, and the calculated correlation coefficients between human and LLM ratings.
- **Correlation Analysis:** As shown in the supplementary scatter plots, we observed significant positive correlations ($\rho = 0.38$ to 0.44, $p < 0.001$), confirming that the LLM judges reliably

track human expert judgment.

- **Systematic Bias:** The data also quantifies a systematic negative bias (LLM scores being lower than human scores by ≈ 0.6). This confirms that our automated evaluation framework acts as a conservative proxy, ensuring that the performance gains reported for RIDE are not inflated by the automated metrics.

To contextualize the use of LLM-based evaluation for baselines not included in blinded physician scoring, we further compared LLM-derived ratings with physician ratings on the sampled subset ($n = 223$). For Qwen3-VL and Med3DVLM, the LLM-based scores showed moderate positive correlations with physician ratings (Spearman's $\rho = 0.38$ and $\rho = 0.44$, respectively; both $p < 0.001$). In addition, the LLM-based evaluation showed a negative mean offset of approximately 0.6 Likert points relative to physician scores, suggesting a generally stricter scoring tendency. These findings indicate that the LLM-based evaluation captures broadly similar rating trends while applying a more conservative scale, and therefore can serve as a supplementary automated reference for large-scale comparison, rather than a replacement for direct physician assessment.

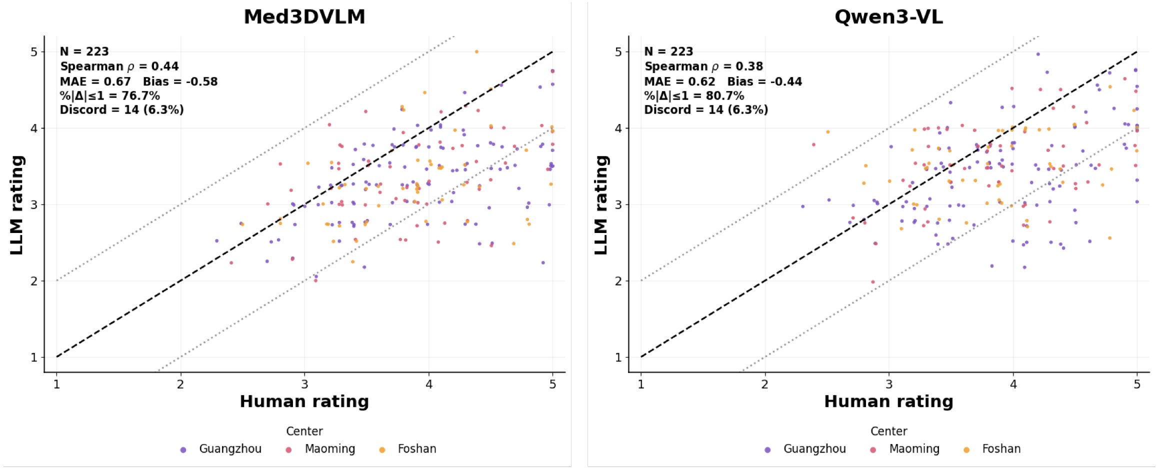

Figure S6: **Case-level relationship between physician ratings and LLM-based ratings on the sampled subset.** Scatter plots show physician ratings (x-axis) and LLM-based ratings (y-axis) for Qwen3-VL and Med3DVLM. Points are colored by center. The black dashed line indicates perfect agreement ($y = x$), and the gray dotted lines indicate a deviation of ± 1 score. Moderate positive correlations were observed for both models (Spearman $\rho = 0.38$ for Qwen3-VL and $\rho = 0.44$ for Med3DVLM), while the LLM-based ratings were generally lower than physician ratings.

Figure S6 shows the relationship between physician ratings and LLM-based ratings for Qwen3-VL and Med3DVLM on the sampled subset. For both models, the LLM-based scores exhibited moderate positive correlation with physician ratings, with Med3DVLM showing a slightly higher Spearman correlation than Qwen3-VL (0.44 vs. 0.38). The overall downward offset of the LLM-based scores relative to physician scores is consistent with a stricter scoring tendency.

S6 TNM Confusion Matrices

Tables S4–S6 summarize clinically salient confusions, including the T2–T3 boundary challenges and M0→M1 false positives.

Table S4: T Stage Confusion Matrix (Row Normalized %)

**Actual Predicted**	**T0**	**T1**	**T2**	**T3**	**T4**
**T0 (n=2)**	38.0%	41.2%	12.6%	4.8%	3.5%
**T1 (n=199)**	1.1%	86.0%	9.3%	2.3%	1.4%
**T2 (n=149)**	0.3%	9.2%	82.7%	5.5%	2.2%
**T3 (n=72)**	0.2%	3.7%	9.1%	79.7%	7.2%
**T4 (n=94)**	0.2%	3.4%	5.3%	10.5%	80.6%

Table S5: N Stage Confusion Matrix (Row Normalized %)

**Actual Predicted**	**N0**	**N1**	**N2**	**N3**
**N0 (n=209)**	76.0%	12.8%	7.0%	4.2%
**N1 (n=49)**	28.0%	43.0%	20.7%	8.2%
**N2 (n=115)**	9.4%	12.7%	56.0%	21.9%
**N3 (n=143)**	6.4%	5.6%	24.4%	63.6%

Table S6: M Stage Confusion Matrix (Row Normalized %)

**Actual Predicted**	**M0**	**M1**
**M0 (n=331)**	79.6%	20.4%
**M1 (n=185)**	30.4%	69.6%

For *N-stage* (Table S5), N1 disease exhibits the lowest per-class recall (43.0%, n=49), with the primary confusion being N1→N0 (28.0%). This is clinically plausible because ipsilateral peribronchial and hilar nodes are often small and may show only mildly increased uptake near the lower limit of reliable PET detection. By contrast, N2 and N3 achieve higher recall (56.0% and 63.6%), as mediastinal and contralateral nodal disease tends to present with more conspicuous metabolic and morphological signatures.

For *T-stage* (Table S4), the dominant off-diagonal mass lies along the T2–T3 boundary (T3→T2: 9.1%; T2→T3: 5.5%), consistent with ambiguity near the 5 cm cutoff, where irregular morphology and partial-volume effects complicate precise size estimation. Large cross-jumping errors (e.g., T1→T4) remain rare ($\leq$3.5%), suggesting preserved ordinal consistency even when exact boundary classification is uncertain.

For *M-stage* (Table S6), the main error mode is M0→M1 overcall (20.4%), often associated with severe granulomatous disease such as active pulmonary tuberculosis, which can mimic metastatic patterns on both CT and PET. We further analyze false-positive and false-negative behavior under modality ablations in the dedicated "False Positive and False Negative Analysis in TNM Staging" subsubsection.

S7 Bootstrap confidence intervals

To quantify uncertainty, we compute case-level bootstrap 95% confidence intervals (CIs) with 1,000 resamples on the test set. For model comparisons, we additionally compute paired bootstrap CIs of metric differences between RIDE and baselines using the same resampled case indices.

Table S7: MDS, LLS, MCS Metrics with 95% Confidence Intervals

Model Name	MDS (95% CI)	LLS (95% CI)	MCS (95% CI)
**Internal Validation** ($n = 244$)			
**RIDE (Ours)**	[89.9, 96.2]	[91.5, 97.1]	[79.9, 89.0]
MedGemma-4B	[67.4, 78.5]	[72.7, 83.1]	[64.8, 76.2]
Qwen3-VL	[65.7, 77.0]	[70.5, 81.2]	[60.5, 72.3]
Qwen2.5-VL	[63.5, 75.1]	[69.2, 80.1]	[55.0, 67.2]
Med3DVLM	[62.2, 73.9]	[71.4, 81.9]	[57.9, 70.0]
MedDr	[61.8, 73.5]	[55.4, 67.6]	[59.2, 71.1]
RadFM-3D	[57.1, 69.2]	[69.6, 80.4]	[48.7, 61.2]
LLaVA-Med	[37.6, 50.1]	[44.5, 57.1]	[25.7, 37.4]
**External Validation** ($n = 276$)			
**RIDE (Ours)**	[86.1, 93.2]	[87.3, 94.1]	[76.8, 85.9]
MedGemma-4B	[61.9, 72.9]	[65.3, 76.0]	[57.8, 69.1]
Qwen3-VL	[60.0, 71.2]	[63.0, 73.9]	[53.4, 64.9]
Qwen2.5-VL	[57.4, 68.8]	[60.8, 71.9]	[49.0, 60.7]
Med3DVLM	[55.6, 67.0]	[63.4, 74.3]	[50.8, 62.5]
MedDr	[56.0, 67.4]	[49.4, 61.0]	[53.0, 64.6]
RadFM-3D	[51.5, 63.2]	[62.6, 73.6]	[42.5, 54.3]
LLaVA-Med	[33.0, 44.4]	[36.1, 47.7]	[20.3, 30.5]

Table S8: T/N/M Staging Accuracy with 95% Confidence Intervals

Model Name	T Stage (95% CI)	N Stage (95% CI)	M Stage (95% CI)
**Internal Validation** ($n = 244$)			
**RIDE (Ours)**	[79.9, 89.0]	[63.9, 75.4]	[72.3, 82.7]
MedGemma-4B	[56.6, 68.8]	[44.5, 57.1]	[52.9, 65.2]
Qwen3-VL	[54.5, 66.8]	[41.7, 54.2]	[50.8, 63.2]
Qwen2.5-VL	[52.9, 65.2]	[38.8, 51.3]	[48.7, 61.2]
Med3DVLM	[51.6, 64.0]	[36.8, 49.2]	[47.8, 60.4]
RadFM-3D	[41.7, 54.2]	[29.2, 41.2]	[45.8, 58.3]
MedDr	[43.7, 56.3]	[28.5, 40.4]	[40.9, 53.4]
LLaVA-Med	[27.3, 39.1]	[13.6, 23.3]	[38.8, 51.3]
**External Validation** ($n = 276$)			
**RIDE (Ours)**	[78.0, 86.9]	[59.3, 70.5]	[70.6, 80.7]
MedGemma-4B	[58.2, 69.4]	[44.3, 56.0]	[55.2, 66.7]
Qwen3-VL	[54.1, 65.6]	[40.0, 51.7]	[52.3, 63.9]
Med3DVLM	[52.3, 63.9]	[38.3, 49.9]	[49.0, 60.7]
Qwen2.5-VL	[51.2, 62.8]	[36.5, 48.1]	[49.0, 60.7]
RadFM-3D	[45.0, 56.8]	[40.0, 51.7]	[49.0, 60.7]
MedDr	[40.0, 51.7]	[32.3, 43.7]	[43.2, 55.0]
LLaVA-Med	[24.7, 35.5]	[17.3, 27.1]	[37.2, 48.8]

Detailed MDS, LLS, and MCS metrics with 95% bootstrap confidence intervals are reported in Supplementary Table S7, and detailed T/N/M staging accuracies with 95% bootstrap confidence intervals are reported in Supplementary Table S8. Supplementary Table S7 shows that the CRCM gains are not only large in point estimate but also stable in interval estimate. For example, on the internal set, RIDE's lower bound exceeds the strongest baseline's upper bound by 11.4 points for MDS, 8.4 points for LLS, and 3.7 points for MCS; the same pattern is preserved on the external set, where the corresponding margins remain 13.2, 11.3, and 7.7 points, respectively. Supplementary Table S8 shows a similar pattern for TNM staging: RIDE's confidence ranges remain above those of the strongest baselines across T-, N-, and M-stage on both internal and external cohorts. The narrowest separation is observed for external N-stage, which remains the most challenging axis, but even there RIDE preserves a 3.3-point margin between its lower bound and the strongest baseline's upper bound, supporting that the staging gains are not confined to a few favorable cases.

S8 Stage Attribution of Errors

We attribute errors to three key stages in the pipeline. Since pipeline errors often cascade (e.g., an anchor failure inherently starves the retrieval module), a single error unit can be attributed to multiple contributing stages simultaneously:

- **Anchor-caused:** Errors originating from the initial Stage I anchors, where key fields (location, presence, SUV classification, metastasis status) were incorrectly identified. If these anchors were incorrect, the final report inherently reflected these mistakes.
- **Retrieval-caused:** Errors resulting from insufficient evidence retrieved during Stage II. These were detected when Stage I anchors were correct but the retrieval phase failed to supply sufficient evidence, resulting in missing or misreported data. We identified such cases by comparing the Stage I anchors with the retrieved evidence slots and checking for coverage.
- **Synthesis-caused:** Errors occurring during Stage III synthesis, where correct anchors and retrieved evidence were used, but the final report still exhibited inconsistencies. This could include contradictions between the anchor and final report, or hallucinated errors where the final report added false positives.

Table S9: Multi-label stage attribution for residual errors in the RIDE framework.

Error Type	Total Units	Anchor-involved (%)	Retrieval-involved (%)	Synthesis-involved (%)
Omission	178	88.2	78.1	11.2
False Positive	84	32.1	48.8	85.7
Mislocalization	63	93.7	61.9	14.3
Misgrading	87	14.9	92.0	68.3
**Overall**	**412**	**62.4**	**73.5**	**40.8**

S9 Synthesis Inconsistency Failure Modes

We further analyze cases where extracted draft units disagree with gold labels on localization, metabolic grade, or malignancy state during the synthesis stage, categorizing errors into six detailed types:

- **(E1) Implicit / non-explicit mention:** e.g., "Other mediastinal regions show no abnormal uptake." (no explicit station ID).
- **(E2) Station format / alias mismatch:** e.g., "Mediastinal nodes (2R, 4L, 4R, station 6) ..." (mixed station formats).
- **(E3) Negation scope error:** e.g., "Multiple cervical nodes are present; PET shows no abnormal focal uptake." (negation scope).
- **(E4) Multi-entity modifier attachment error:** e.g., "Stations 2R/4R/7 enlarged; only 7 is hypermetabolic." (modifier attachment).
- **(E5) Physiologic uptake confounding:** e.g., "Symmetric uptake in fat spaces (SUV_{max} 7.5)." (physiologic uptake).
- **(E6) Numeric parsing / unit formatting error:** e.g., "Mass $2.4 \times 2.2 \times 2.5$ cm; SUV_{max} $\approx$14.8." (numeric formatting).

Table S10: Failure mode distribution. Percentages sum to 100% over synthesis error cases.

Failure type	Proportion among errors (%)
E1 Implicit / non-explicit mention	28.0
E2 Station format / alias mismatch	21.0
E3 Negation scope error	17.0
E4 Multi-entity modifier attachment error	14.0
E5 Physiologic uptake confounding	12.0
E6 Numeric parsing / unit formatting error	8.0

S10 Inference Time and Resource Consumption

Inference Time: For each test sample, the total inference time is approximately 28 seconds, divided as follows: Preprocessing (5 seconds), Inference/model forward pass (18 seconds), and Post-processing (5 seconds). This processing time is consistent across the test set, demonstrating the model's efficiency for real-time applications in clinical settings.

GPU Memory Usage: The RIDE model requires approximately 64GB of GPU memory during inference on the Nvidia H100. Breakdown: Input Data (PET+CT) takes 10GB, Model Parameters (Network) takes 40GB, Intermediate Layers take 12GB, and Post-processing buffers take 2GB. This level of GPU memory usage is typical for large-scale multi-modal frameworks, and its memory consumption is directly proportional to its performance, making it suitable for environments with access to high-performance computing hardware.

Table S11: GPU Memory Usage Breakdown for RIDE Model on Nvidia H100.

**Component**	**Memory Usage (GB)**
Input Data (PET+CT)	10
Model Parameters (Network)	40
Intermediate Layers	12
Post-processing buffers	2
**Total GPU Memory Usage**	**64**

S11 Standard Linguistic Evaluation Metrics Formulation

To evaluate the lexical and syntactic similarity of generated drafts against reference reports, we utilize the following standard Natural Language Generation (NLG) metrics.

BLEU computes the n-gram overlap precision:

$$\mathrm{BLEU}_n = BP \cdot \exp\left(\sum_{k=1}^{n} w_k \log p_k\right) \tag{S1}$$

where BP is the brevity penalty, p_k is the clipped k-gram precision, and w_k is the weight.

METEOR calculates a weighted harmonic mean of unigram precision (P) and recall (R):

$$\mathrm{METEOR} = F_{\mathrm{mean}} \cdot \left(1 - 0.5 \cdot \left(\frac{ch}{m}\right)^3\right) \tag{S2}$$

where F_{mean} is the harmonic mean, ch is the number of chunks, and m is the number of matches.

ROUGE-L evaluates content coverage based on the longest common subsequence (LCS):

$$\mathrm{ROUGE}_L = \frac{(1+\beta^2)\, R_{\mathrm{lcs}}\, P_{\mathrm{lcs}}}{R_{\mathrm{lcs}} + \beta^2 P_{\mathrm{lcs}}} \tag{S3}$$

where R_{lcs} and P_{lcs} are LCS-based recall and precision.

CIDEr gauges consensus-based semantic similarity using TF-IDF weighted n-gram matching:

$$\mathrm{CIDEr} = \frac{1}{m} \sum_{j=1}^{m} \frac{\mathbf{g}(c) \cdot \mathbf{g}(r_j)}{\|\mathbf{g}(c)\|\, \|\mathbf{g}(r_j)\|} \tag{S4}$$

where $\mathbf{g}(\cdot)$ is the TF-IDF vector, c is the candidate text, and r_j are reference texts.

S12 Implementation Detail and work example

To rigorously evaluate the generated drafts without manual bias, the free-text reports are processed using a deterministic Natural Language Processing (NLP) pipeline. To ensure transparency and

reproducibility, we detail the NLP architecture, the terminology mapping strategy, and provide a step-by-step worked example based on a translated representative whole-body PET/CT clinical report from our cohort.

S12.1 NLP Pipeline and Terminology Mapping

The extraction framework relies on established clinical NLP techniques to parse unstructured text into the structured units required for CRCM calculation:

1. **Syntactic Parsing and Dependency Extraction:** We utilize the Stanford Stanza toolkit [?], with biomedical and clinical model packages specifically trained for radiology text [?], to perform tokenization, part-of-speech tagging, and universal dependency parsing. This constructs a grammatical tree that links anatomical entities with their respective descriptive modifiers (e.g., size, SUVmax, and morphological traits).
2. **Anatomical Synonym Mapping via RadLex Ontology:** To resolve vocabulary variations, extracted anatomical entities are normalized using a dictionary aligned with the RadLex radiology lexicon [?], following established practices for multi-institutional radiology information extraction [?]. Terms are mapped to our predefined 21 staging-relevant region keys. For instance, the variations "station 2R", "right upper paratracheal node", and "level 2R" are deterministically mapped to the canonical key `Paratracheal`. "Right upper lobe" and "RUL" map to `Right_upper_lobe`.
3. **Metabolic Discretization and Negation Resolution:** SUVmax values attached to anatomical nodes in the dependency tree are discretized into Grade 0 (< 2.5), Grade 1 ($2.5 \leq \text{SUVmax} \leq 10$), and Grade 2 ($> 10$),following the widely adopted clinical threshold for separating benign from malignant FDG uptake [?]. For qualitative descriptions lacking numeric values, we implement a customized NegEx-based rule set [?] to resolve negation scopes. For example, "no obvious uptake" or "metabolism is unremarkable" triggers a benign state (Grade 0 / Level 0), whereas "hypermetabolic" defaults to Grade 1 or 2 depending on morphological context.

S12.2 Step-by-Step Metric Computation

Below, we sample three key sentences from a processed clinical report to demonstrate how the parsed units are matched against the gold standard to compute the Lesion Localization Score (LLS), Metabolic Detection Score (MDS), and Malignancy Classification Score (MCS).

Sentence 1 (Focus on LLS - Anatomical Localization Mapping)
Generated Text: *"Multiple lymph nodes are seen in the right hilar and mediastinal (stations 2R, 4R) regions, the largest measuring 1.2cm × 1.1cm, showing increased glucose metabolism, SUVmax approximately 4.0."*

- **NLP Extraction:** The dependency parser detects a conjunction of spatial entities. The RadLex mapper normalizes "station 2R" to `Paratracheal`, "station 4R" to `Lower_paratracheal`,

and “right hilar” to `Hilar`.

- **Matching:** The predicted draft generates these three distinct nodal region keys. The gold-standard set $\mathcal{U}$ confirms the presence of hypermetabolic disease in exactly these three stations.

- **LLS Calculation:** Since the exact region keys match the gold standard without conflating adjacent stations (e.g., the model did not hallucinate station 7 / `Subcarinal`), the localization micro-accuracy (LLS) for these units scores 1.

Sentence 2 (Focus on MDS - Metabolic Quantification)
Generated Text: *“A mass-like soft tissue density is observed in the posterior segment of the right upper lobe... showing increased FDG uptake, SUVmax approximately 8.5.”*

- **NLP Extraction:** The region key maps to `Right_upper_lobe`. The dependency tree links the numerical token “8.5” to the term “SUVmax”.

- **Discretization:** According to our 0-2 scale, 8.5 falls into the intermediate tier ($2.5 \leq \text{SUVmax} \leq 10$). Thus, the predicted metabolic grade is **Grade 1**.

- **MDS Calculation:** The expert gold-label also records this specific mass as Grade 1. Because the predicted grade strictly matches the gold standard, it yields an MDS of 1 for this unit.

Sentence 3 (Focus on MCS - Malignancy Risk via Negation)
Generated Text: *“A strip-like high-density shadow with clear boundaries is seen in the lingular segment of the left upper lobe, with no obvious metabolism.”*

- **NLP Extraction:** The region key maps to `Left_upper_lobe`. The NegEx module detects the negation trigger (“no obvious”) governing the target (“metabolism”), and the parser identifies benign morphological cues (“clear boundaries”, “strip-like”).

- **State Assignment:** The malignancy state is predicted as **Level 0** (benign/absent).

- **MCS Calculation:** The expert gold-standard confirms this is a benign post-inflammatory fibrotic strip (Level 0). The risk stratification matches, resulting in an MCS of 1. If an unconstrained baseline LLM hallucinated this as a “metastatic nodule” due to contextual cross-organ noise, the MCS for this unit would severely drop to 0.

S12.3 Limitations of the CRCM NLP Extraction Tool: Failure Case Analysis

While our deterministic NLP pipeline (utilizing Stanford Stanza, RadLex, and NegEx) enables scalable extraction of structured units for CRCM computation, free-text clinical reports occasionally contain complex syntactic structures or atypical formatting that fall outside the pipeline’s deterministic rules.

To systematically audit the reliability of our evaluation metric, we executed the NLP extraction tool across the entire $N = 831$ cohort and compared the outputs against the established expert

gold-standard labels. As detailed in the hierarchical audit in Table S12, the NLP tool exhibited perfect extraction agreement in 713 cases (85.8%). In the remaining 118 cases, we identified a total of 245 specific region-attribute units (out of over 12,000 extracted units cohort-wide) that disagreed with the gold standard.

Table S12: **Hierarchical Audit of CRCM NLP Extraction Limitations.** The table illustrates the discrepancy funnel from the cohort level ($N = 831$ cases) down to the unit level ($n = 245$ discrepant units). The bottom section provides a multi-label root cause analysis of the specific parser failure modes on these 245 units.

Audit Level	Metric / Category	Count (n)
**Unit-Level**	Estimated Total Region-Attribute Units	>12,000
	Total Discrepant Units to Audit	245
**Failure Modes**	E1: Implicit / non-explicit mention coverage	84
	E2: Station format / nested alias mismatch	62
	E3: Negation scope error (NegEx limit)	55
	E4: Multi-entity modifier attachment error	44
	E5: Physiologic uptake confounding	35
	E6: Numeric parsing / Regex formatting error	25

Counts denote the number of the 245 discrepant units exhibiting each error type.
Because a single complex sentence can trigger multiple failure modes simultaneously, the counts sum to more than 245.

S12.4 Multi-Label Root Cause Analysis

We conducted a rigorous post-hoc manual review of all 245 discrepant units to categorize the NLP extraction limitations (Table S12, bottom section). Because a single complex finding can trigger multiple parsing failures simultaneously, these modes are treated as multi-label attributes:

- **(E1) Implicit / Non-Explicit Mention Coverage:** If a report states, *"Other mediastinal regions show no abnormal uptake"*, the parser fails to explicitly map this to specific target stations like `Paratracheal`, resulting in a false omission.
- **(E2) Station Format / Nested Alias Mismatch:** In nested enumerations like *"Mediastinal nodes (2R, 4L, 4R)..."*, the parser might fail to attach the "mediastinal node" context to the naked alphanumeric tokens "2R" and "4L".
- **(E3) Negation Scope Error (NegEx limitation):** In multi-clause boundaries, such as *"Multiple cervical nodes are present; however, PET shows no abnormal focal uptake"*, the parser may incorrectly flag the nodes as positive due to syntactic distance.
- **(E4) Multi-Entity Modifier Attachment Error (Stanza limitation):** In compound sentences like *"Stations 2R, 4R, and 7 are enlarged, but only 7 is hypermetabolic"*, the dependency tree might erroneously propagate the "hypermetabolic" state to all three stations.
- **(E5) Physiologic Uptake Confounding:** If a report notes *"Intense uptake in the brown fat spaces ($SUV_{\max}$ 7.5)"*, the pipeline extracts the high $\text{SUV}_{\max}$ and falsely assigns a malignant state, failing to understand the benign semantic context.

- **(E6) Numeric Parsing / Regex Formatting Error:** Typographical anomalies, such as *"$SUV_{\max}$ 14.8"* or *"SUVmxa 14.8"*, cause the regular expression extractors to miss the quantitative value.

S12.5 Metric Reliability Confirmation

Despite these inherent parser limitations, a formal evaluation on the test set yielded a Cohen's κ of 0.88 for anatomical localization (LLS), 0.85 for metabolic grading (MDS), and 0.82 for malignancy classification (MCS). This "almost perfect agreement" (Landis & Koch criteria) confirms that while the NLP tool has known systematic limitations (E1-E6), it remains a highly equitable, objective, and scalable comparative metric for benchmarking generative models.

S13 Comparison against 3D-native baselines

Table S13: **3D-vs-3D comparison: TNM staging accuracy.** All models receive the same complete 3D volumetric input. 95% bootstrap CIs are reported to quantify uncertainty.

	**Internal Validation** ($n = 244$)			**External Validation** ($n = 276$)		
Model (3D input)	T Stage (95% CI)	N Stage (95% CI)	M Stage (95% CI)	T Stage (95% CI)	N Stage (95% CI)	M Stage (95% CI)
**RIDE (Ours)**	[79.9, 89.0]	[63.9, 75.4]	[72.3, 82.7]	[78.0, 86.9]	[59.3, 70.5]	[70.6, 80.7]
Med3DVLM	[51.6, 64.0]	[36.8, 49.2]	[47.8, 60.4]	[52.3, 63.9]	[38.3, 49.9]	[49.0, 60.7]
RadFM-3D	[41.7, 54.2]	[29.2, 41.2]	[45.8, 58.3]	[45.0, 56.8]	[40.0, 51.7]	[49.0, 60.7]
LLaVA-Med	[27.3, 39.1]	[13.6, 23.3]	[38.8, 51.3]	[24.7, 35.5]	[17.3, 27.1]	[37.2, 48.8]

Table S14: **3D-vs-3D comparison: Clinical report quality (MDS / LLS / MCS).** All models receive the same complete 3D volumetric input. 95% bootstrap CIs are reported to quantify uncertainty.

	**Internal Validation** ($n = 244$)			**External Validation** ($n = 276$)		
Model (3D input)	MDS (95% CI)	LLS (95% CI)	MCS (95% CI)	MDS (95% CI)	LLS (95% CI)	MCS (95% CI)
**RIDE (Ours)**	[89.9, 96.2]	[91.5, 97.1]	[79.9, 89.0]	[86.1, 93.2]	[87.3, 94.1]	[76.8, 85.9]
Med3DVLM	[62.2, 73.9]	[71.4, 81.9]	[57.9, 70.0]	[55.6, 67.0]	[63.4, 74.3]	[50.8, 62.5]
RadFM-3D	[57.1, 69.2]	[69.6, 80.4]	[48.7, 61.2]	[51.5, 63.2]	[62.6, 73.6]	[42.5, 54.3]
LLaVA-Med	[37.6, 50.1]	[44.5, 57.1]	[25.7, 37.4]	[33.0, 44.4]	[36.1, 47.7]	[20.3, 30.5]
