# Supplementary figures and images for: TNM-Accountable Whole-Body 3-Dimensional Fluorodeoxyglucose Positron Emission Tomography/Computed Tomography Report Drafting in Lung Cancer Cohorts via Structured Impressions and Organ-wise Exemplar Synthesis
Source: Research (Wash D C). 2026 Jul 21;9:1343. doi: 10.34133/research.1343 (PMC13385537; doi:10.34133/research.1343)

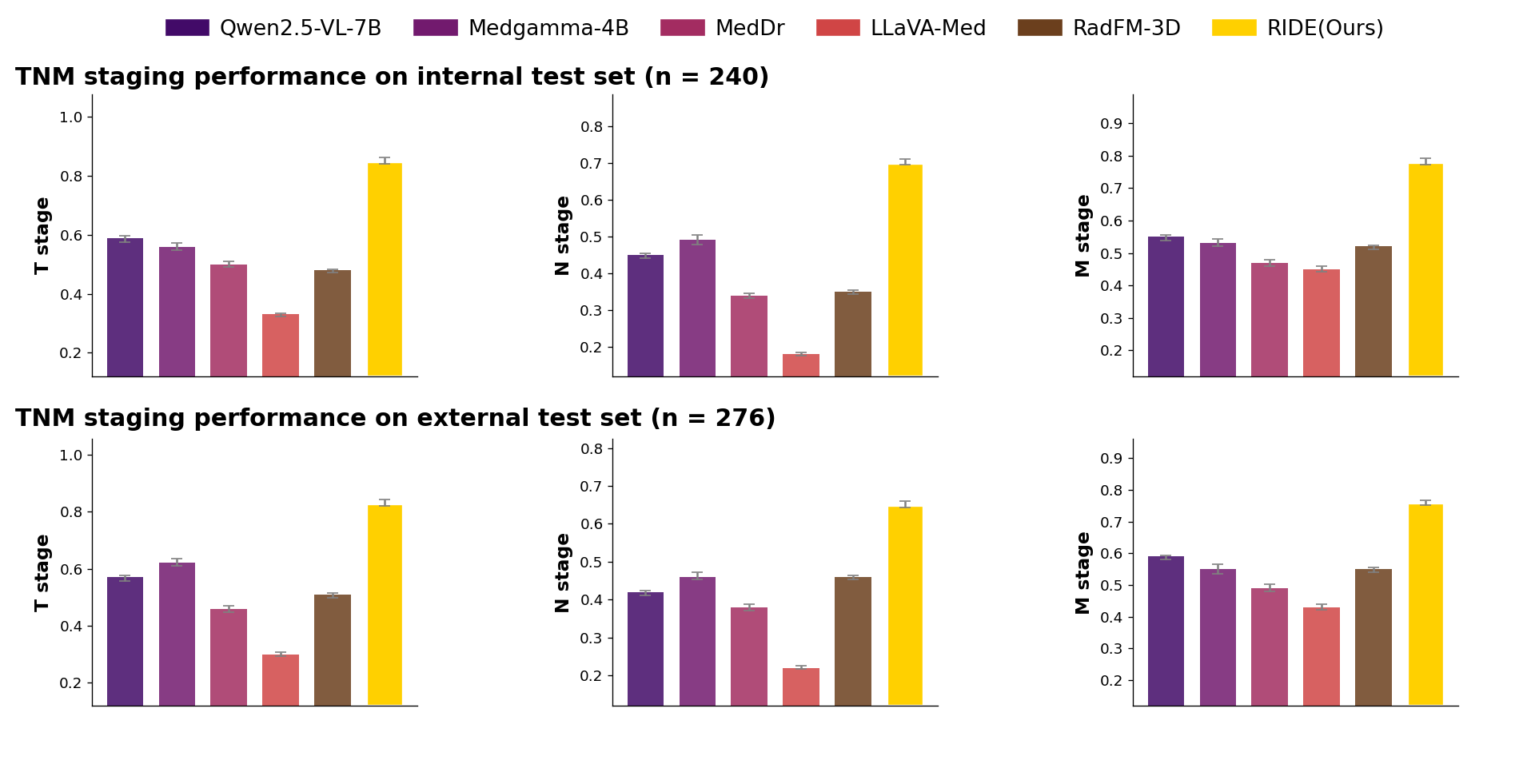

Supplement: Supplementary 1 — Supplementary Text Figs. S1 to S7 Tables S1 to S14 [file research.1343.f1.zip › S1.png]

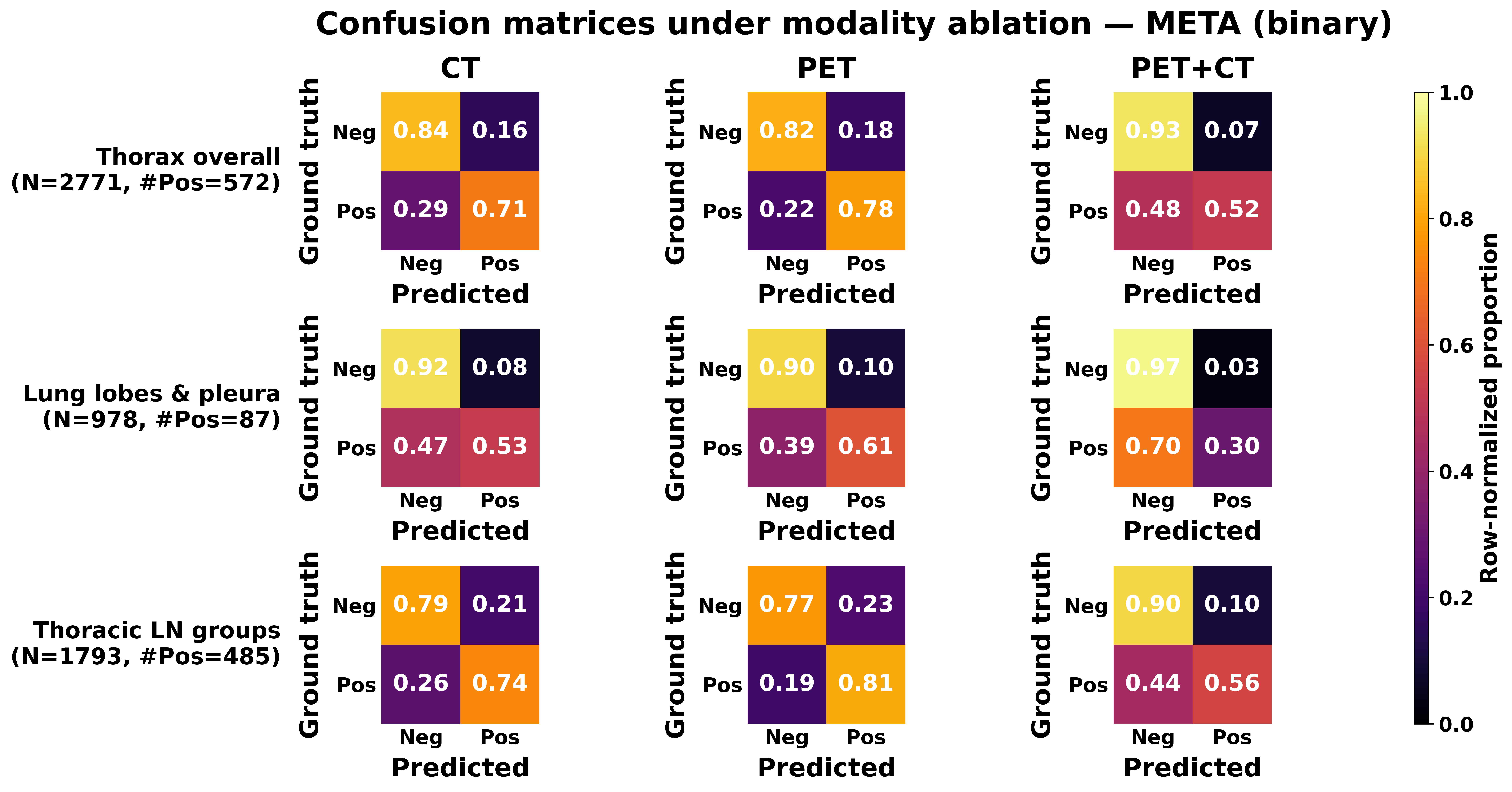

Supplement: Supplementary 1 — Supplementary Text Figs. S1 to S7 Tables S1 to S14 [file research.1343.f1.zip › S2.png]

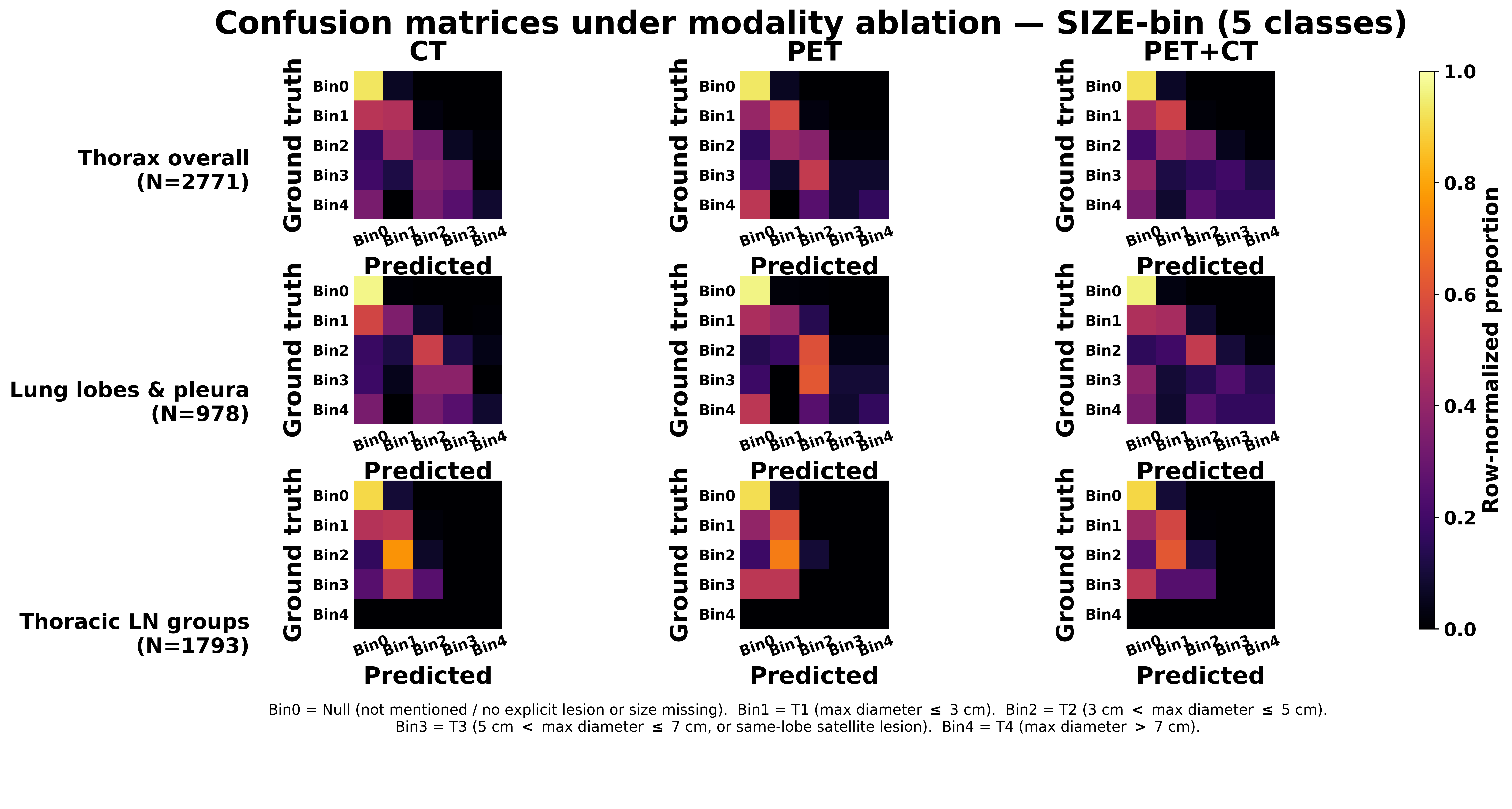

Supplement: Supplementary 1 — Supplementary Text Figs. S1 to S7 Tables S1 to S14 [file research.1343.f1.zip › S3.png]

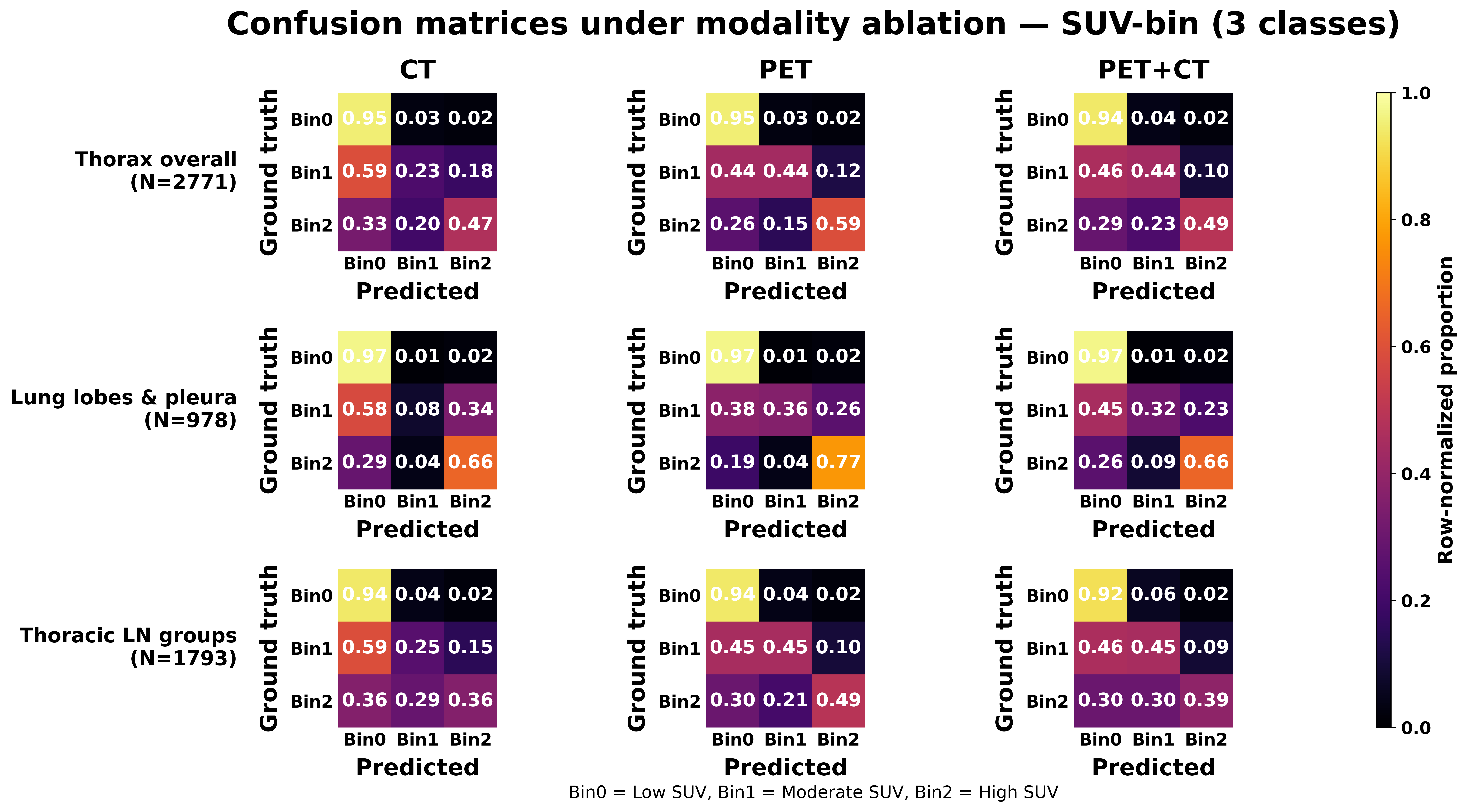

Supplement: Supplementary 1 — Supplementary Text Figs. S1 to S7 Tables S1 to S14 [file research.1343.f1.zip › S4.png]

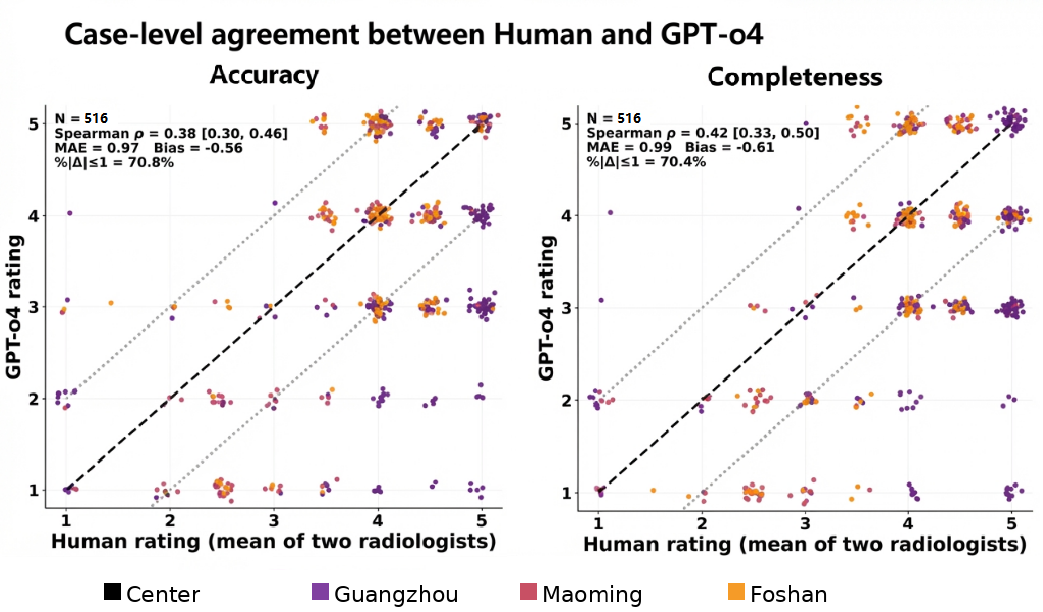

Supplement: Supplementary 1 — Supplementary Text Figs. S1 to S7 Tables S1 to S14 [file research.1343.f1.zip › S5.png]

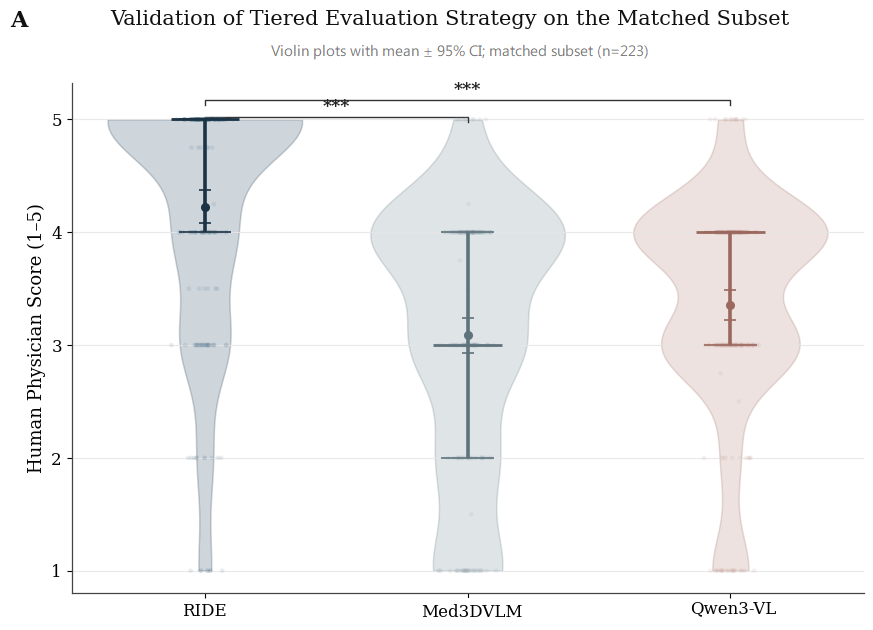

Supplement: Supplementary 1 — Supplementary Text Figs. S1 to S7 Tables S1 to S14 [file research.1343.f1.zip › S6.png]

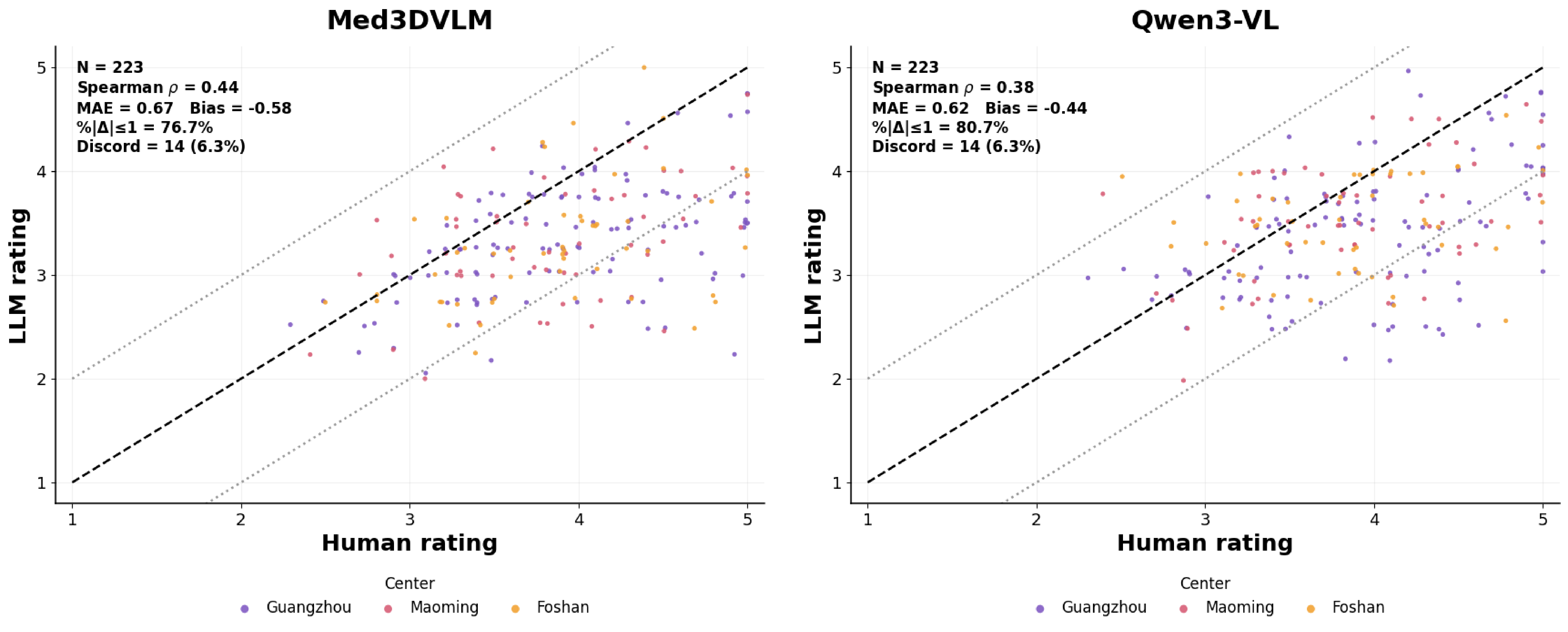

Supplement: Supplementary 1 — Supplementary Text Figs. S1 to S7 Tables S1 to S14 [file research.1343.f1.zip › S7.png]
